# Deciphering the controlling factors for phase transitions in zeolitic imidazolate frameworks

**DOI:** 10.1093/nsr/nwae023

**Published:** 2024-01-13

**Authors:** Tao Du, Shanwu Li, Sudheer Ganisetti, Mathieu Bauchy, Yuanzheng Yue, Morten M Smedskjaer

**Affiliations:** Department of Chemistry and Bioscience, Aalborg University, Aalborg 9220, Denmark; Department of Mechanical Engineering-Engineering Mechanics, Michigan Technological University, Houghton MI 49931, USA; Department of Chemistry and Bioscience, Aalborg University, Aalborg 9220, Denmark; Physics of AmoRphous and Inorganic Solids Laboratory (PARISlab), Department of Civil and Environmental Engineering, University of California, Los Angeles, Los Angeles, CA 90095, USA; Department of Chemistry and Bioscience, Aalborg University, Aalborg 9220, Denmark; Department of Chemistry and Bioscience, Aalborg University, Aalborg 9220, Denmark

**Keywords:** metal-organic frameworks, phase transitions, melting, glass formation, transferable deep learning force field, ring orientation

## Abstract

Zeolitic imidazolate frameworks (ZIFs) feature complex phase transitions, including polymorphism, melting, vitrification, and polyamorphism. Experimentally probing their structural evolution during transitions involving amorphous phases is a significant challenge, especially at the medium-range length scale. To overcome this challenge, here we first train a deep learning-based force field to identify the structural characteristics of both crystalline and non-crystalline ZIF phases. This allows us to reproduce the structural evolution trend during the melting of crystals and formation of ZIF glasses at various length scales with an accuracy comparable to that of *ab initio* molecular dynamics, yet at a much lower computational cost. Based on this approach, we propose a new structural descriptor, namely, the ring orientation index, to capture the propensity for crystallization of ZIF-4 (Zn(Im)_2_, Im = C_3_H_3_N_2_^−^) glasses, as well as for the formation of ZIF-zni (Zn(Im)_2_) out of the high-density amorphous phase. This crystal formation process is a result of the reorientation of imidazole rings by sacrificing the order of the structure around the zinc-centered tetrahedra. The outcomes of this work are useful for studying phase transitions in other metal-organic frameworks (MOFs) and may thus guide the development of MOF glasses.

## Teaser

A new structural descriptor, the ring orientation index, is proposed as a governing factor for phase transitions in zeolitic imidazolate frameworks (ZIFs), facilitating the design of ZIF glasses for various applications.

## INTRODUCTION

Metal-organic frameworks (MOFs) are porous materials made up of metal ions and organic ligands. The tunable pore structure and chemical compositions of MOFs make them promising materials for various applications such as gas sorption and separation, shock absorption, catalysis, and ion transportation [1–3]. MOFs can undergo thermo-induced phase transitions, including the formation of MOF glasses through mechanical vitrification [4], synergistic stimuli [5], and melt-quenching of their crystalline counterpart. MOF glasses have attracted a great deal of attention as they are free of grain boundaries (e.g. advantageous for battery applications and functional thin films [6]) and moldable, while (partially) inheriting the porous structure of the parent crystals. Among the types of MOF crystals that can be transformed into a glassy state, a large fraction of the studies are based on zeolitic imidazolate frameworks (ZIFs) [7,8]. Not all crystalline MOFs can be transformed into a glass due to the lack of a stable liquid state, since the melting temperature is above the decomposition temperature [9]. ZIFs are based on metal ions such as Zn^2+^ or Co^2+^ connected by imidazolate-based ligands, giving rise to tetrahedral structural units that further assemble into larger medium-range order (MRO) network topologies. Various studies on melting and glass-formation of ZIFs have been conducted. For example, the ability of ZIFs to retain porosity in the melt-quenched glass state is correlated with the MRO structural features [10], while the appearance of the low-density state during the melting process of ZIF-4 is caused by the ligands-induced exchange behavior [11]. Additionally, the significant decrease in glass transition temperature (*T*_g_) of ZIF-62 (Zn(Im)_2-x_(bIm)_x_, where Im is imidazole, C_3_H_3_N_2_^−^, and bIm is benzimidazole, C_7_H_5_N_2_^−^) under isostatic pressure is ascribed to the MRO structure change upon densification [12]. The high glass forming ability of ZIF-62 is correlated with the high steric hindrance and frustrated network dynamics as well as the soft and flexible nature of the structure [13]. Recently, through high-temperature *in situ* nuclear magnetic resonance experiments, crystalline ZIF-62 is proposed to undergo progressive ‘softening’ with increasing temperature [14], while previous work has ascribed melting to Zn−N bond breaking [7,9]. Therefore, a deep understanding of how the structure evolves during melting is still needed and necessary for the discovery of new glass-forming MOFs with tailored functionalities [15].

In addition to their temperature dependence, the framework structure of crystalline MOFs features complex behaviors in their response to pressure. That is, external high pressure can induce structural transformations, such as pressure-induced crystal-to-crystal transition and pressure-induced amorphization, leading to the formation of a denser phase [16,17]. While the pressure-induced crystal-to-crystal transition is only observed in a small fraction of MOFs [17], most MOFs exhibit pressure-induced amorphization, for instance, the prototypical ZIF-8 (Zn(mIm)_2_, where mIm is 2-methylimidazolate (C_4_H_5_N_2_^−^)) undergoes irreversible amorphization under compression at 0.3 GPa [18]. Such pressure-induced transition of ZIF-8 involves a first amorphization caused by the disruption of the MRO structure and a second densification due to the change in short-range order structure [19]. The denser ZIF-4 (Zn(Im)_2_) exhibits a reversible amorphization at a slightly higher hydrostatic pressure of 0.35 GPa [20]. This pressure-induced amorphization of ZIFs is correlated with mechanical instability due to pressure-induced elastic softening [21]. By controlling temperature and pressure, ZIF-4 can undergo several phase transitions, including amorphous-to-amorphous transition (i.e. low-density to high-density amorphous phases) and the formation and melting of a denser crystalline phase (ZIF-zni) [7].

It is well-known that the preparation conditions, including thermal and pressure history, influence the formation of traditional oxide glasses [22,23]. For instance, the low- to high-density amorphous transition of silica glass is triggered by a sequence of percolation transitions upon pressurization [24]. Such pressure sensitivity can also be observed in some MOF systems. In ZIFs, high pressure offers a new route to synthesize glasses since it stabilizes the ZIF liquid state at lower temperatures [25], and the glass transition temperature (*T*_g_) of ZIF-62 glass is significantly lowered when melt is quenched under a modest pressure of 60 MPa [12]. This pressure history dependence of glass transition in ZIF-62 is found to be highly correlated with the medium-range structure, i.e. structural features present at length scales of around 5–20 Å. Despite the progress in understanding the crystalline structure transformations of MOFs under high-temperature and high-pressure conditions, the different non-crystalline phases under such extreme environments are challenging to be identified and characterized due to the experimental limitations of, e.g. diffraction measurements [15].

To understand the phase transitions in MOFs, molecular dynamics (MD) simulations have been demonstrated to be an effective method for studying different MOF systems at varying length and time scales [26–28]. Previous works show that *ab initio* MD simulations can accurately capture the melting behavior of crystalline MOFs and generate the configurations of melt-quenched glasses [27,29,30]. However, such first principle simulations are limited to small systems and short timescales due to the large computational cost, making it challenging to capture the structural features at the medium-range scale. Classical MD simulations with empirical force fields are routinely applied to crystalline MOFs, but since they cannot offer a realistic estimation of the activation energy associated with bond formation and breakage, they are difficult to be applied to non-crystalline MOFs [31]. Reactive force fields with the ability to describe dynamical bonding may offer a trade-off between *ab initio* and classical MD simulations [32], but recent work has shown that they cannot capture all the necessary structural details in the case of ZIFs, including the ring structures [28]. A computationally efficient, yet accurate force field is thus needed to fully understand the phase transitions in ZIFs.

Thanks to the capability of machine learning (ML) in revealing complex mapping patterns in high-dimensional data, ML-based force fields (MLFFs) have shown great promise in simulating various chemical reactions. This is because they can efficiently describe the potential energy surface with an accuracy comparable to *ab initio* MD (AIMD) simulations, although most MLFFs cannot distinguish the different components of the energy terms (i.e. bond energy, van der Waals interaction, Coulombic potential, etc.) [33,34]. Typical models for MLFF may be categorized into either kernel regression or neural network depending on the complexity of the model [35,36]. The accuracy of MLFF largely relies on the selection of training data as generated from expensive AIMD simulations. This dataset should cover a large portion of phase space for the simulated systems. MLFFs based on neural networks have been extensively applied in simulating various properties of crystalline MOF phases, such as mechanical and diffusional properties [37,38]. MLFFs have also been successfully applied in the simulation of structural transformations [39], solid-liquid phase transitions [40], and amorphization processes of systems such as oxides, chalcogenides, and metals [41–43]. We note that two other MLFFs for ZIF glasses have recently been developed [11,44], but they are not parametrized for ZIF systems under high pressures.

To better understand the phase transitions in ZIFs, and especially the medium-range structure of ZIF phases under different temperature-pressure conditions, here we propose the following strategy. First, we train a deep learning-based force field (DLFF) using the DeePMD method for the melt-quenching simulations of ZIF-4 [45]. We focus on ZIF-4 because of its flexible phase transitions [46], simple ligand chemistry, and good glass-forming ability [20]. We use the DeePMD method because of its transferability and relatively low computational cost. The training datasets for the DLFF are generated from AIMD simulations of different ZIF materials and other related substances containing fewer elements to ensure that the DLFF is transferable to other ZIFs. We then perform MD simulations of the melt-quenching process of ZIF-4 under varying pressures. The degree of disorder of ZIF-4 at different stages of melting is quantified by the local structure of the metal cations as well as the orientation of imidazole rings. The DLFF approach allows us to identify the latter as an important new structural characteristic, inspired by work on glassy silica [47], which involves the stereographic projection of the imidazole ring and accurately captures the structural disorder at the medium-range length scale. Based on these findings, we are thus able to quantify the synergistic high-pressure and high-temperature effects on the structural vitrification of ZIF-4. While we focus on ZIF-4, we also discuss how the outcomes can be transferable to characterizing phase transitions in other ZIFs and MOFs.

## RESULTS

### Force field training and validation

**Figure 1.**
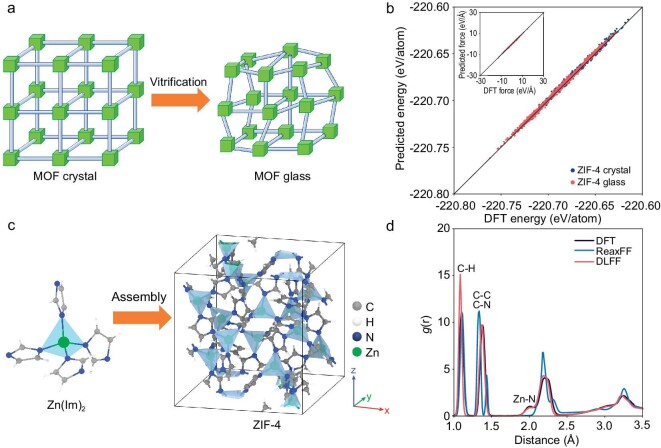
Reproduction of the structure and atomic interactions of ZIF-4 using deep learning force field. (a) Basic unit of MOF crystal consisting of metal nodes (green cubes) connected by organic linkers (blue rods), with a MOF glass being formed by introducing topological disorder. (b) Comparison of the energy and atomic forces of the unit cell of ZIF-4 crystal and glass calculated by DFT and predicted by the present DLFF. Note that the datasets of ZIF-4 crystal and ZIF-4 glass are from the AIMD trajectory. The root-mean square errors (RMSEs) of energy and force prediction for ZIF-4 crystal are 9.5×10^−4^ eV and 5.3×10^−2^ eV/Å, respectively, and for ZIF-4 glass they are 1.8×10^−3^ eV and 1.5×10^−1^ eV/Å, respectively. (c) Schematic of basic unit and the unit cell structure of ZIF-4. (d) Pair distribution functions *g* (*r*) of ZIF-4 crystal simulated using DFT, ReaxFF, and DLFF, respectively.

To describe the formation mechanism of MOF glasses (here, ZIF-4), it is crucial that the force field accurately accounts for the interatomic interactions in both ordered and disordered structures (see Fig. 1a). To this end, we select a series of configurations from the trajectories of AIMD simulations including both the crystalline and disordered structures of different ZIFs (i.e. ZIF-4, ZIF-62, ZIF-8, and SALEM-2 (Zn(Im)_2_)), and other substances with fewer elements than ZIFs (e.g. imidazole, benzene, methane, and zinc nitride). An overview of the training structures is presented in Figs S1 and S2. To further explore the potential energy surface, an active ML approach using DP-GEN is applied to expand the training set to increase the accuracy of the force field [48]. DP-GEN automatically collects new datasets by iteratively comparing the quantities calculated by density functional theory (DFT) and DLFF on-the-fly, thereby increasing the transferability of the resulting DLFF. The expanded datasets were collected by the configurations at high-temperature and high-pressure conditions, ensuring that the DLFF covers the potential energy surface of the subsequent simulations. We also observe that further increasing the datasets will not influence the simulation results, which further verifies the completeness of the DFT-based datasets (Fig. S3). The detailed process of the DLFF training is given in the Supplementary Methods section.

According to the predicted energy and forces (inset figure) shown in Fig. 1b, the resulting DLFF can well reproduce the interatomic interactions within the ZIF-4 configurations. We further validate the ability of the DLFF in reproducing the atomic structure of ZIF-4 crystal. As shown in Fig. 1c, ZIF-4 is a cag topology (named after CaGa_2_O_4_ (variscite)) with periodically distributed Zn-centered tetrahedra linked to four imidazolate ligands. The pore structure of ZIF-4 is evident with periodic channels surrounded by imidazole rings. Figure 1d illustrates the pair distribution functions (PDFs) of ZIF-4 crystal at 300 K, as simulated using DFT (i.e. AIMD), ReaxFF, and the present DLFF. The PDFs indicate that the new DLFF outperforms ReaxFF in reproducing the structure as it well captures the structure predicted by the DFT simulations. The simulated X-ray differential correlation function *D*(*r*) of ZIF-4 glass using DLFF matches well with the corresponding experimental data regarding the peak positions at 1.4 Å, 2.0 Å, 3.0 Å, and 6.0 Å, corresponding to the C-C/C-N, Zn-N(1st), Zn-N(2nd), and Zn-Zn distances, respectively (Fig. S4). The DLFF-simulated N−Zn−N bond angle distribution of ZIF-4 crystal also agrees well with the DFT simulated one. In contrast, ReaxFF shows a significant deficiency (Fig. S5), as reported in Ref [28].

### Melt-quenching simulations

**Figure 2.**
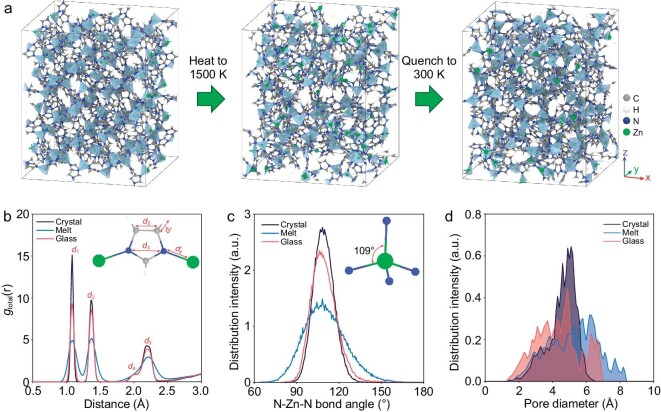
Melt-quenching simulation of ZIF-4 at zero pressure using deep learning force field. (a) Atomic configurations of ZIF-4 at different stages of the melt-quenching process, i.e. ZIF-4 crystal, molten ZIF-4 at 1500 K, and quenched ZIF-4 glass at 300 K. (b–d) Calculated (b) pair distribution functions, (c) N−Zn−N bond angle distribution functions, and (d) pore size distribution of the ZIF-4 crystal, melt (at 1500 K), and glass.

We now use the trained DLFF to simulate the melt-quenching of ZIF-4 to generate its glass structure. As shown in Fig. 2a, the initially ordered structure of crystalline ZIF-4 becomes disordered upon melt-quenching, i.e. the perfectly aligned Zn-tetrahedra become distorted and partially undercoordinated. The PDFs of the three different stages, i.e. crystal, melt, and glass, are shown in Fig. 2b. The three main peaks located at around 1.1, 1.4, and 2.2 Å exhibit an initial widening and then narrowing trend upon melt-quenching, indicating that the glass structure is closer to that of the crystal compared to the melt structure. In addition to the three main peaks, we find that the shoulder peak at around 2.0 Å disappears in the molten state but reappears in the glass state, as the Zn−N coordination bonds break upon melting (also reflected in the Zn−N partial PDF shown in Fig. S6) [9]. The partial Zn−N pair distribution function shown in Fig. S6 indicates that most of the Zn−N bonds persist during the melt-quenching process. Meanwhile, the molten state of ZIF-4 is confirmed by the intersection of the first two peaks of the partial PDF of Zn−N due to the presence of nitrogen exchanges between the first and second coordination shells of the zinc cations, in agreement with previous AIMD results [29]. We then investigate the tetrahedra structure by calculating the N−Zn−N bond angle (Fig. 2c). The unimodal distribution of the N−Zn−N bond angle at 109° indicates that most tetrahedra persist upon melt-quenching, while the peak first becomes broader and then narrower upon melt-quenching, in agreement with the AIMD results reported in Ref [27]. Given the importance of porosity in MOF glasses, we finally investigate the capability of the DLFF in simulating the pore structure of ZIF-4 upon melt-quenching. As shown in Fig. 2d, there are two distinct cavity sizes of around 3.0 and 5.0 Å, respectively, in ZIF-4 crystal. The ZIF-4 glass exhibits a broader distribution of pore size, indicating a more disordered structure that is in agreement with the experimental findings [49].

### Simulated melting under different pressures

**Figure 3.**
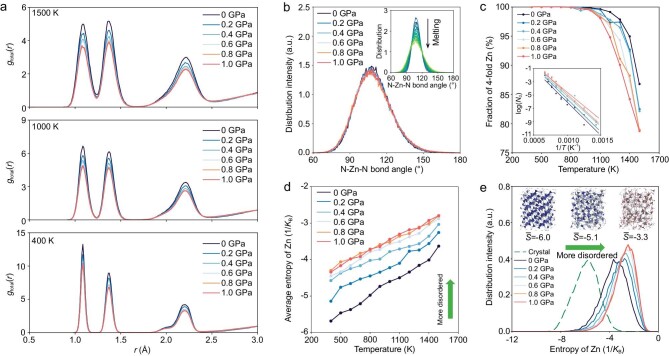
Melting process of ZIF-4 crystal at varying pressures. (a) Pair distribution functions of ZIF-4 during the melting process at 400, 1000, and 1500 K. (b) N−Zn−N bond angle distribution of ZIF-4 at 1500 K under varying pressures (0 to 1.0 GPa). Inset: N−Zn−N bond angle distribution at zero pressure under varying temperatures (300 to 1500 K), with the change in color from dark blue to light green representing an increase in temperature. (c) Fraction of 4-fold Zn in ZIF-4 upon increasing temperature at varying pressures. Inset: Arrhenius fit of coordination defects formation as explained in the text. (d) Average entropy of Zn atoms during the ZIF-4 crystal melting process as a function of temperature at varying pressures. (e) Distribution of entropy of Zn atoms in ZIF-4 melted at 1500 K at varying pressures (green dashed line: entropy distribution of ZIF-4 crystal equilibrated at 300 K for comparison). Insets: tetrahedra configurations colored by the entropy of the central Zn atoms for the ZIF-4 crystal (left), glass (middle), and melt (right), with the blue-to-red color change representing an increase in entropy.

To understand the pressure-induced structural changes in ZIF-4 glasses, we then apply a hydrostatic pressure of up to 1 GPa during melt-quenching. We first consider the PDFs of ZIF-4 under varying pressures upon heating (Fig. 3a). At all three temperatures, all three main peaks become broader with increasing pressure, which echoes the experimentally observed pressure-induced amorphization in ZIF-4 since the full width at half-maximum of the PDF peaks represents the fluctuation of interatomic distances, correlated with the degree of structural disorder [25,50]. The same trend is also observed in the Zn-N partial PDFs, and the melting states of all the samples are confirmed by the overlapped peaks at 1500 K (Fig. S7). By calculating the potential of mean force for the Zn-N coordination at 1500 K and varying pressures (Fig. S8), we find that the free energy barrier associated with the Zn−N bond breakage decreases with an increase in the pressure, indicating that the network structure becomes softer and more prone to melt upon densification. These findings agree well with the experimental and AIMD results reported in Ref [25]. Furthermore, the N−Zn−N bond angle distribution of ZIF-4 slightly widens upon densification (Fig. 3b), whereas this distribution significantly widens upon heating (inset of Fig. 3b). This pressure-induced bond angle widening is more pronounced at lower temperatures (Fig. S9).

All Zn cations are tetrahedrally coordinated with imidazole ligands in the ZIF-4 crystal, as depicted in the low-temperature region of Fig. 3c. At 900 K, the fraction of four-fold coordinated Zn cations begins to decrease upon heating and the final fraction in the molten state at 1500 K further decreases with an increase in pressure. Specifically, there are around 86% four-fold Zn at 1500 K and zero pressure, while the fraction is only around 79% at a pressure of 1.0 GPa. As shown in the inset of Fig. 3c, the fraction of defective Zn cations ${N}_d$ (i.e. under- or over-coordinated Zn) under different pressures exhibit an Arrhenius temperature dependence: ${N}_d \propto {\mathrm{exp}}( { - {E}_a/RT} )$ as described in Ref [27]. The activation energy ${E}_a\ $at zero pressure is around 83 kJ mol^−1^, while it gradually decreases to 69 kJ mol^−1^ at 1.0 GPa pressure. The pressure-induced decrease in ${E}_a\ $of defective Zn formation can be attributed to the decrease in the energy barrier that is associated with the breakage of the Zn−N bond (Fig. S8).

**Figure 4.**
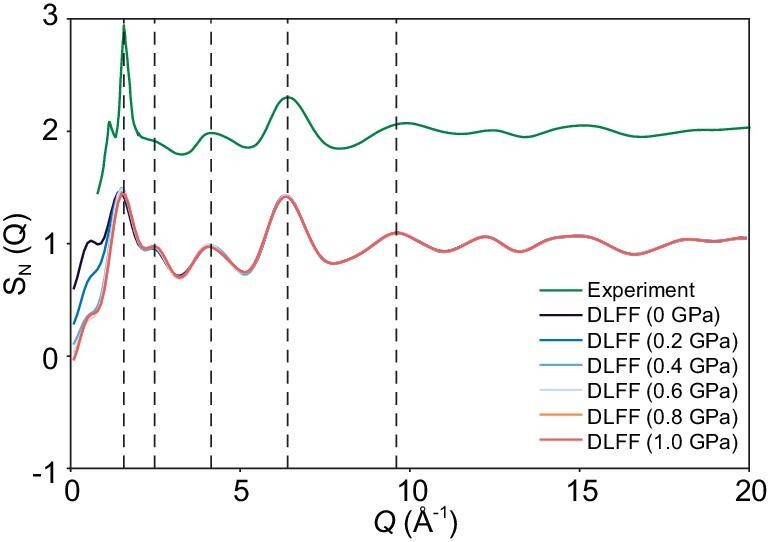
Neutron structure factors (*S*_N_(*Q*)) of ZIF-4 glasses. Comparison between experimental (top) and simulated (bottom) neutron structure factor of ZIF-4 glasses under varying pressures. The experimental curve is for the ambient pressure glass and obtained from Ref [9].

To further quantify the change in the local structural environment of Zn cations with temperature and pressure, we have calculated the local entropy of each Zn cation in ZIF-4. Note that here the entropy is not the exact entropy, but instead the excess entropy per atom based on a series of interatomic correlation functions. Since the two-body excess entropy accounts for ∼90% of the configurational entropy, we have calculated the local entropy based on the transformation of the PDF for individual atoms (see Eq. 1 in the Supplementary Methods section). Note that, the value of entropy is usually negative since it is relative to the ideal gas which has the maximal value. Therefore, a smaller (more negative) value of entropy represents a more ordered structure. This metric has been extensively used to characterize the ordered and disordered environments in heterogeneous systems [51]. As shown in Fig. S10, the entropy of Zn can indeed be used to distinguish the different states of ZIF-4 and different crystalline structures of Zn(Im)_2_. As expected, the averaged entropy of Zn in all the cases increases monotonically with temperature due to the increase in the degree of disorder (Fig. 3d). We also find that the entropy of Zn in ZIF-4 increases upon pressurization. Furthermore, the entropy distributions of Zn at 1500 K (Fig. 3e) and in quenched glass state (Fig. S11) exhibit a similar dependence on pressure, suggesting a pressure-induced disordering of the Zn local structure relative to the structure of the crystalline ZIF-4. The MRO structure of ZIF-4 glass is investigated by calculating the neutron structure factor (*S_N_*(*Q*)). As shown in Fig. 4, the main peaks of *S_N_*(*Q*) show good agreement with the experimentally obtained ones. The gradual shift of the first sharp diffraction peak (∼1.57 Å^−1^) towards a larger *Q* value in the structure factor with pressure confirms the densification of the MRO structure.

### Imidazole ring orientation as a structural descriptor

**Figure 5.**
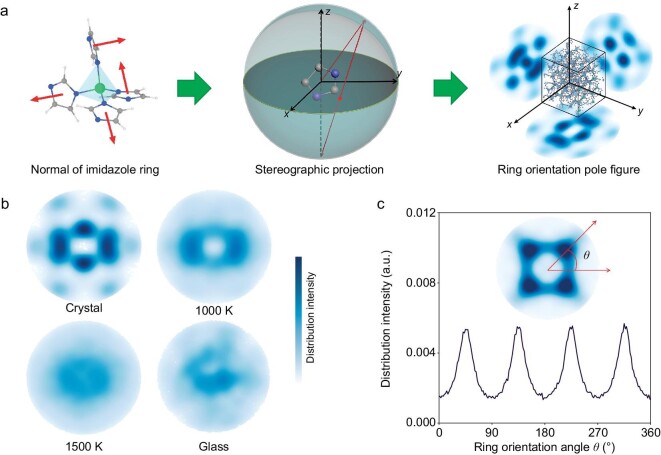
Orientation of imidazole rings in ZIF-4 at different states during melt-quenching. (a) Schematic of the calculation of the ring orientation pole figure, including the identification of the imidazole ring, calculation of the normal of the imidazole ring, and projection of the normal of all the imidazole rings to the three planes using stereographic projection. (b) Pole figures of ring orientation projected on the *yz*-plane of ZIF-4 crystal at zero pressure, melted at 1000 K, melted at 1500 K, and the melt-quenched glass. (c) Distribution histogram of the orientation angle *θ* as quantified from the pole figure of SALEM-2 projected on the *yz*-plane.

While the obtained DLFF has been found to well reproduce the pressure-induced disordering of ZIF-4 at different temperatures, these analyses focused on the local structure of Zn and cannot fully explain the structural difference between the crystalline and glassy states. For example, ZIF-zni crystal and ZIF-4 glass exhibit a similar PDF and entropy of Zn (Fig. S12). We therefore shift our focus to the role of the organic linker, i.e. the imidazole ring. Specifically, we introduce a new structural descriptor to describe ZIF phases based on a stereographic projection to quantify the orientation degree of the normal of the imidazole rings. Figure 5a shows the procedure to calculate the pole figure of imidazole ring orientation. First, we identify all the imidazole rings within the ZIF-4 structure to calculate the normal of the imidazole rings. Then, each orientation vector is normalized and stereographically projected on the three planes (i.e. *x* = 0, *y* = 0, and *z* = 0, corresponding to the *yz*-, *xz*-, and *xy*-planes). The orientation pole figure is obtained as the density histogram of the stereographic projections of all the ring normals. The detailed calculation procedure is given in the Supplementary Methods section.

Figure 5b shows that the orientation pole figure of ZIF-4 strikingly depends on the temperature during the melt-quenching process. For the ZIF-4 crystal, the orientation of imidazole rings exhibits an expected directional preference as observed from the distribution concentration in the pole figure. Upon melting, the distribution of the pole figure becomes more uniform, indicating a more random distribution of the ring orientations as expected for a more disordered structure at a higher temperature. Additional pole figures as a function of temperature during melting are provided in Figs S13 and S14. Interestingly, we also observe a reordering of the ring orientation upon quenching, since the pole figure of the glass is less uniform than that at 1500 K. As such, the orientation of imidazole rings is a descriptor of the structural order. We further analyze the pole figures by calculating the distribution intensity as a function of the orientation angle in the projected plane (see Fig. 5c). As SALEM-2 crystal is a sodalite topology with ordered ring orientation, the distribution histogram of its orientation angle shows that the four main orientation angles are found at 45°, 135°, 225°, and 315°, respectively. The ring orientation of SALEM-2 also exhibits an isotropic behavior as seen from the similar distributions in all three projected planes (Fig. S15). From the orientation distribution histograms at different temperatures, we observe that the ring orientation of ZIF-4 becomes more random upon melting (Fig. S16).

### Pressure-induced reorientation of imidazole rings

**Figure 6.**
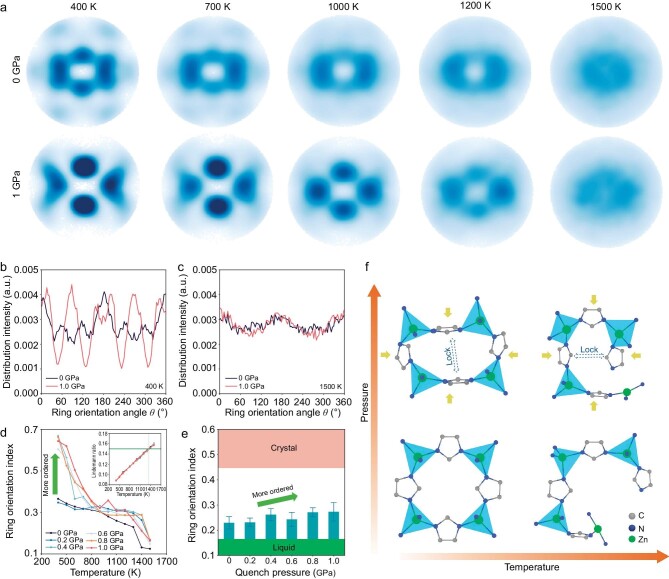
Orientation of imidazole rings in ZIF-4 during melt-quenching. (a) Orientation pole figures of ZIF-4 at 400 K, 700 K, 1000 K, 1200 K, and 1500 K for zero pressure (top panel) and 1 GPa pressure (bottom panel) during the heating process. (b and c) Orientation distribution histograms of imidazole ring in ZIF-4 at (b) 400 K and (c) 1500 K projected in the *yz*-plane. (d) Evolution of ring orientation index (ROI) of ZIF-4 at varying pressures as a function of temperature. Inset: Zn-N Lindemann ratios for ZIF-4 phases at varying pressures during the heating process as a function of temperature. (e) ROI value of ZIF-4 glass as a function of applied pressure. Error bar is obtained from ten MD-simulated structures with different initial temperature profiles. ROI value of the crystal is calculated as the minimum ROI of ten ZIF-4 crystal structures relaxed at 300 K and zero pressure, while ROI value of the liquid is obtained as the maximum ROI of ten ZIF-4 molten structures at zero pressure. (f) Schematic of ZIF-4 structure transformations with temperature and pressure. H atoms and two imidazole groups of each Zn atom are omitted for clarity.

The concept of ring orientation allows us to analyze the medium-range structure of ZIF-4 during the melt-quenching process under varying pressures. Figure 6a depicts the orientation pole figures of ZIF-4 crystal during the melting process at zero and 1.0 GPa pressure, respectively. Although the pole figures become increasingly diffuse upon heating under both pressures, we can still observe a more ordered structure in the pressurized ZIF-4, especially at a lower temperature. The pressure-induced ordering does not depend on the selected system size, as similar orientation pole figures of ZIF-4 are found for system sizes of 2176 and 17 408 atoms as shown in Fig. S17. The distribution histograms of ring orientation at 400 and 1500 K are shown in Figs. 6b and c, respectively. Based on the observation that the distribution profiles at 1 GPa are more fluctuating than those at 0 GPa, we infer that the ring orientation becomes more anisotropic with pressure. We also calculate the ring orientation index (ROI) of each system as a function of temperature during melting (Fig. 6d). The ROI value is calculated based on the roughness of the distribution histogram of the orientation angle *θ* as quantified from the pole figure (see Supplementary Methods section for more details). ROI decreases with increasing temperature irrespective of the pressure conditions, indicating that this new structural feature characterizes the vitrification behavior of ZIF-4. The ring orientations become more anisotropic upon pressurization, except at 0.2 GPa, and thus the ROI is slightly lower than that at zero pressure. This phenomenon indicates that the threshold pressure for reorientating the imidazole rings in ZIF-4 is between 0.2 and 0.4 GPa when *T* <700 K, as also confirmed from the pole figure shown in Fig. S14.

To further characterize the melting process, we have calculated the generalized Lindemann ratio [52] as a function of temperature based on the width of the first peak in the Zn-N partial PDF (inset of Fig. 6d). The Lindemann ratio does not have a clear dependence on the pressure as all the samples exhibit a similar melting point of around 1350 K (according to the criterion of 15% used, e.g. in Ref [9]), further confirming that all the samples reach the molten state at 1500 K. Interestingly, ROI decreases significantly at the melting point (Fig. 6d), indicating that the anisotropic-to-isotropic transformation of ring orientation is a sign of the liquid phase formation. Next, we determine ROI values of the resulting glasses obtained upon melt-quenching. As shown in Fig. 6e, the ring orientation in the resulting glass structure also exhibits a clear dependence on pressure, with a more anisotropic ring orientation at higher pressure. For comparison, the ROI values of ZIF-4 liquid and crystal are around 0.16 and 0.45, respectively, suggesting that high pressure induces a more ordered ring orientation that closely resembles that of the crystal. By isolating the effect of temperature and pressure, the pressure-induced ring ordering can be attributed to the closer interaction between imidazole rings that block the rotation (Fig. 6f).

### Describing ZIF structures using entropy and ring orientation

Our findings point to the existence of two distinct effects of pressure on the ZIF-4 structure. First, for the Zn cations, the application of pressure during melt-quenching affects both the strength of the Zn−N coordination bonds and the N−Zn−N bond angle within the Zn-centered tetrahedra, leading to a disordered local structure of Zn cations, agreeing with the findings reported in Ref [7]. Second, while the structure of the imidazole ring consists of relatively strong covalent bonds that are hard to break during melting, the orientation of these rings becomes more anisotropic with pressure. This enables the formation of a densified structure with reduced porosity (Figs S18 and S19), indicating the driving force of the anisotropic ring orientation is the minimization of atomic volume. According to experimental observations [53], the transition of ZIF-4 to other high-pressure polymorphs (such as ZIF-zni) occurs via a reconstructive process with Zn−N bond breaking. Although it is not feasible to simulate the recrystallization of high-pressure polymorphs due to the limited time scale of MD simulation (even with the present DLFF), the mechanism proposed in this work can account for the reorientation process of imidazole rings prior to recrystallization by sacrificing the ordered structure around the Zn-centered tetrahedra.

**Figure 7.**
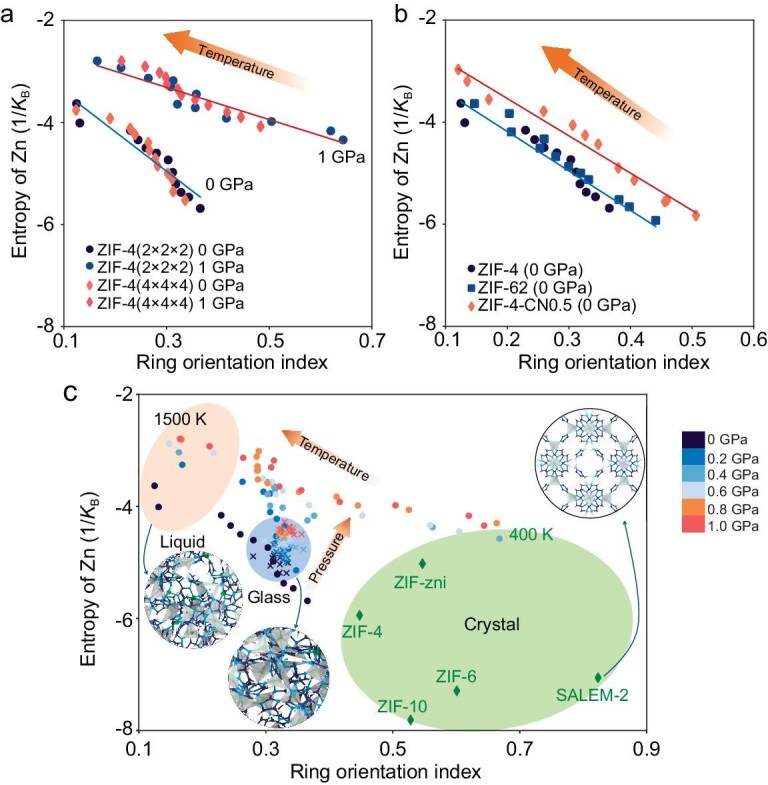
Correlation between ring orientation index and entropy of Zn in various ZIF phases. (a and b) Effect of (a) system size (2176 vs. 17 408 atoms, where *N* × *N* × *N* represents the initial system generated by *N* replications of the ZIF-4 unit cell in all directions) and (b) chemical linkers on the correlation between ring orientation index and entropy of Zn in the ZIF system. The lines are to guide the eye. (c) Different crystalline ZIFs with composition of Zn(Im)_2_ and ZIF-4 melted at different temperatures and pressures. Spheres with different colors represent different temperatures and pressures. Crosses represent the melt-quenched glasses under different temperatures. Diamonds represent the crystalline polymorphs of Zn(Im)_2_ (i.e. ZIF-4, ZIF-6, ZIF-10, ZIF-zni, and SALEM-2) for comparison.

Finally, we compare the two structural fingerprints, i.e. (1) the entropy of Zn to characterize the short-range structure and (2) the ROI to characterize the medium-range structure. As shown in Fig. 7a, both structural fingerprints are robust to the selected system size. When extrapolated to other ZIF materials, i.e. ZIF-4 with other functionalized imidazolate linkers, the two structural fingerprints can still be used to characterize the disordering process upon melting (Fig. 7b). We then correlate the entropy of Zn with ROI to distinguish the different structures of ZIF-4 upon melt-quenching under various pressures (Fig. 7c). Crystalline ZIF phases exhibit a large value of ROI and small entropy of Zn, indicating a highly ordered structure of both the Zn-centered tetrahedra and imidazole rings. Upon melting, the liquid phases of ZIF-4 exhibit a disordered structure, both in terms of the tetrahedra and imidazole rings, as these phases are located at the upper left corner of the plot. The glass structures are located between the crystalline and liquid phases, reflecting the results shown in Fig. 2. We find that pressurization drives the ZIF-4 structure towards a state of higher entropy and ROI, indicating a counterbalance effect between pressure-induced vitrification and crystallization [25,53]. Although ZIF-zni and SALEM-2 crystals (with the same composition as ZIF-4) exhibit distinct values of entropy and ROI compared to ZIF-4 (see green region in Fig. 7c), the structural order of these crystals is captured by the entropy-ROI plot. That is, SALEM-2 exhibits the most ordered structure, while ZIF-4 crystals feature a structure that most resembles that of ZIF-4 glasses.

## CONCLUSION

The combined structural fingerprints of ROI and entropy enable a classification of the different ZIF structures, from glassy to crystalline structure. This approach is not restricted to ZIF systems, as it can easily be extended to other MOFs to reveal their structure-property relationships. Here, by combining the entropy of the Zn-based tetrahedra with the ROI, we conclude that pressure plays two distinct roles in controlling the ZIF structure. That is, the Zn-centered tetrahedra become more disordered upon pressurization, while the orientation of the imidazole ring becomes more ordered. Although the pressure-induced ring ordering at high temperature resembles the polymorphic transformation into ZIF-zni phase, the simulation time scale does not allow the entire crystallization process to be captured, but both the low-density to high-density transition (at zero pressure) and initial crystallization of ZIF-zni (at low temperature and high pressure) are well described. In addition to the isostatic pressure used herein, we expect the use of non-isostatic pressures (e.g. shear) to be a potential route for tuning the ring orientation. Although the stereographic projection-based structural feature cannot currently be directly determined from experiments, this structural descriptor should be highly correlated with the heterogeneity of the glass structure, which can in turn be characterized using mechanical and spectroscopy measurements. Finally, we note that since the developed DLFF is found to be transferable to other functionalized derivatives of ZIF systems, it can be used to determine the properties of new ZIF glasses prior to their synthesis. The DLFF file and training datasets provided by this study can also be used for subsequent research. If necessary, additional DFT-based datasets can be incorporated to generate a new DLFF with extended applications.

## METHODS

### MD simulations with DLFF

All the MD simulations rely on the deep learning-based force field (DLFF) trained in this work, and the detailed training process can be found in the Supplementary Information. The melt-quenching simulations of ZIF-4 under various pressures were performed using the Large-scale Atomic/Molecular Massively Parallel Simulator (LAMMPS) integrated with the DeePMD-kit package [45,54]. The melt-quench procedure followed that in our previous work with slight modifications [26]. We selected a series of pressures (i.e. 0, 0.2, 0.4, 0.6, 0.8, and 1.0 GPa) for densification of ZIF-4 structure.

The structure was first subjected to an energy minimization process and relaxed at 10 K for 7.5 ps using a Berendsen thermostat. The system was then heated to 300 K in 1.25 ps and equilibrated at 300 K and zero pressure in the *NPT* ensemble for 12.5 ps to obtain the crystal configuration using the Nosé–Hoover thermostat/barostat. The system was further heated to 1500 K in 50 ps and equilibrated at 1500 K and the designated pressure for another 50 ps, which was long enough to ensure the system to be fully melted. The melted configurations were subsequently quenched to 300 K under the designated pressure at a moderate cooling rate of 5 K/ps to achieve the glass configuration, since the cooling rate (except 0.1 K/ps) does not significantly influence the structure of the resulting glass (Fig. S20). The insensitivity to cooling rate of ZIF glasses may be attributed to the large imidazolate ligands with a high steric hindrance, which have extremely sluggish diffusion kinetics in the liquid state [55]. Note that slow cooling rates such as 0.1 K/ps will increase the risk of breaking imidazole rings as the melting (*T*_m_) and decomposition temperature (*T*_d_) of ZIF-4 measured in argon are 863 K and 875 K, respectively [56]. This small difference between *T*_d_ and *T*_m_ made the imidazole rings prone to break near the simulated melting temperature. Consistent with previous AIMD simulations [57], thermal decomposition was also observed in our simulations when using a cooling rate of 0.1 K/ps, i.e. this simulated ZIF-4 glass has a small number of broken rings (around 4%). Therefore, to mitigate the thermal decomposition, we applied a relatively fast cooling rate (i.e. 5 K/ps) to prepare the ZIF-4 glasses. The accuracy and generalizability of the DLFF were verified by the structure simulation of other crystalline phases of Zn(Im)_2_ and functional-derived phases of ZIF-4 [58].

### Structural analyses

The structures of ZIF-4 during melt-quenching, resulting glasses, and crystals were analyzed in terms of the pair distribution function (PDF), bond angle distribution function, neutron structure factor *S*(*Q*), pore size distribution, ring statistics, etc. The PDFs were obtained by calculating the distances between all the atom pairs into distribution histograms. The coordination number of Zn atom was then determined using a cutoff of 3.0 Å, which corresponds to the minimum after the first peak in the Zn−N partial PDF. The distribution of N−Zn−N bond angle was calculated based on the identified Zn−N coordination bonds. The neutron *S*(*Q*) was calculated through the Fourier transform of the PDF using the Faber-Ziman formalism, following the method in Ref [12]. The pore structure was characterized using the Zeo++ package [59] with a probe size of 0.5 Å. The ring statistics were analyzed using the RINGS package [60], where the imidazole rings were determined using Guttman's shortest paths criterion [61] that consists of 3 C and 2 N atoms.

The local entropy of Zn cation and ROI are used to quantify the degree of disorder of Zn tetrahedra and imidazole rings, respectively. The ROI value of the imidazole rings was calculated based on the stereographic projection method [47], which enables the mapping of the three-dimensional vectors at the surface of a sphere onto a plane. The detailed steps to calculate local entropy and ROI can be found in the Supplementary Information.

## Supplementary Material

nwae023_Supplemental_File

## Data Availability

The files of the deep learning force field, related training dataset, and the LAMMPS input sample are available on GitHub https://github.com/OxideGlassGroupAAU/DeepZIF.

## CODE AVAILABILITY

CP2K, LAMMPS, DeePMD, and OVITO are freely available at https://www.cp2k.org, https://www.lammps.org, https://deepmodeling.com, and https://www.ovito.org, respectively.
