# Supplementary material for: Deciphering the controlling factors for phase transitions in zeolitic imidazolate frameworks
Source: Natl Sci Rev. 2024 Jan 13;11(4):nwae023. doi: 10.1093/nsr/nwae023 (PMC10980346; doi:10.1093/nsr/nwae023)
Supplement: nwae023_Supplemental_File [file nwae023_supplemental_file.pdf]

Supplementary Materials for

Deciphering the Controlling Factors for Phase Transitions in Zeolitic Imidazolate Frameworks

Tao Du[1], Shanwu Li[2], Sudheer Ganisetti[1], Mathieu Bauchy[3], Yuanzheng Yue[1], Morten M. Smedskjaer[1,*]

[1]*Department of Chemistry and Bioscience, Aalborg University, Aalborg East 9220, Denmark*

[2]*Department of Mechanical Engineering-Engineering Mechanics, Michigan Technological University, MI 49931, USA*

[3]*Physics of AmoRphous and Inorganic Solids Laboratory (PARISlab), Department of Civil and Environmental Engineering, University of California, Los Angeles, CA 90095, USA*

* Corresponding author. E-mail: mos@bio.aau.dk

This PDF file includes:

Supplementary Text

Figs. S1 to S27

Tables S1 to S2

Supplementary Text

Ab initio MD simulations. The data set for training the deep learning potential were first generated using *ab initio* MD simulations following the procedure employed in Ref. [1]. The input files for *ab initio* MD simulations are adapted from the Supplementary file in Ref. [1]. A series of systems containing C, H, N, and Zn elements were selected as the training sets, including both the crystalline and disordered structures of ZIF-4, SALEM-2, ZIF-8, ZIF-62, elementary substances, and compounds. Note that, two different compositions of ZIF-62 are selected, i.e., ZIF-62-0.5 and ZIF-62-0.25, corresponding to $Zn(Im)_{1.5}(bIm)_{0.5}$ and $Zn(Im)_{1.75}(bIm)_{0.25}$, respectively. An overview of the systems used for generating the data set is provided in Supplementary Table S1. All the *ab initio* calculations were carried out at the DFT level [2] using the Quickstep module of the CP2K package[3] with the hybrid Gaussian and plane wave method (GPW) [4]. As suggested in Ref. [5], the basis functions are mapped onto a multi-grid system with the default number of four different grids with a plane-wave cutoff for the electronic density to be 600 Ry, and a relative cutoff of 40 Ry to ensure the computational accuracy. The AIMD trajectories at elevated temperatures were obtained in the *NVT* ensemble with a timestep of 0.5 fs. The temperature was controlled using the Nosé–Hoover thermostat [6]. The exchange-correlation energy was calculated using the Perdew-Burke-Ernzerhof (PBE) approximation [7], and the dispersion interactions were handled by utilizing the empirical dispersion correction (D3) from Grimme [8]. The pseudo potential GTH-PBE combined with the corresponding basis sets DZVP-MOLOPT-SR-GTH were employed to describe the valence electrons [9].

Deep learning force field construction. The deep learning force field was constructed using the DeePMD-kit package. The training dataset consists of two parts: (1) the initial training data from the trajectory of AIMD simulations and the single energy calculation of the crystalline structures

(i.e., ZIF-4, SALEM-2, ZIF-8, ZIF-62-0.5) under deformations; and (2) the expanded dataset realized by single energy calculation using the active machine learning method implemented in the DP-GEN package [10]. The detailed constituents and configurations of training data are summarized in Supplementary Fig. S1 and S2 and Supplementary Tables S1 and S2. The test set is adopted from the trajectory of AIMD simulations of ZIF-62-0.25. We employed a commonly used network structure [11,12] for deep learning as follows: (1) the embedding network containing 3 layers with the number of neurons per layer equal to 25, 50, and 100, respectively; (2) the fitting network containing 3 layers with the number of neurons per layer all equal to 240. The descriptor "se_e2_a", which contains both angular and radial information of atomic configurations, was used as the model of force field. The training process included two processes, i.e., the initial training and final training. Both processes included 6,000,000 steps of iterations. The loss function for the training process included the energy, force, and virial terms. For the initial training, the learning rate dynamically changed from 1e-3 to 1e-9. The atomic interaction beyond 0.6 Å were treated using the ZBL repulsive interactions to avoid the overlap of atoms at high temperatures, and the prefactors of the energy, force, and virial terms dynamically changed from 0.2 to 1, 500 to 1, and 0.02 to 0.2, respectively. The final training process removed the ZBL interaction and the prefactors of all terms were set to 1 as the learning rate changed from 1e-5 to 1e-8.

We also tried the descriptor "se_e2_r", which only included the radial information of atomic configurations to train the model. However, the resulting force field cannot reproduce the glass structure as well as the descriptor "se_e2_a" (Supplementary Fig. S21a). We also tried different cutoffs for interatomic interaction (i.e., 5.0, 6.0, 7.0 Å), but the simulation result is not significantly influenced by the cutoffs (Supplementary Fig. S21b). Following the literature results, a cutoff for interatomic interaction was set to be 6 Å to achieve a balance between accuracy and

computational efficiency. The comparisons between the results of energy, force, and stress obtained with the optimized DP model and the AIMD calculations are provided in Supplementary Figs. S22-S24.

More details of MD simulations. The initial configuration of the ZIF-4 crystal was obtained from the Cambridge Structural Database [13], which was further replicated into a 2×2×2 supercell of 2,176 atoms. This system size is commonly used in the MD simulations of ZIF glasses using ReaxFF when studying the structural features upon glass formation [14,15]. To exclude the possibility of significant size effects in this work, we also attempted the use of a much larger system size with a 4×4×4 supercell of 17,408 atoms. However, the resulting glass structures did not exhibit a significant dependence on the system size (Supplementary Fig. S25) and we therefore use systems with 2,176 atoms in the remainder of this work. Other crystalline phases of $Zn(Im)_2$ (i.e., SALEM-2, ZIF-zni, ZIF-6, and ZIF-10) are dynamically equilibrated under 300 K and zero pressure for 12.5 ps before structural analysis. The transferability of this DLFF is verified by simulating the newly discovered ZIF glasses with the cyano-functionalized imidazolate linkers [16]. The structural properties and comparison with experiments confirm the accuracy and generalizability of the DLFF (Supplementary Fig. S26).

Note that the thermodynamic and structural properties of the structure at each state were analyzed based on the trajectory equilibrated at the specified temperature for 50 ps in the *NVT* ensemble with a dump interval of 0.25 ps to ensure proper statistical averaging. During the simulation, periodical boundary conditions (PBC) were applied in all directions. A timestep of 0.25 fs was used for the motion integration. The configurations obtained from the simulations were visualized using the OVITO package [17].

Local entropy of Zn atoms. Since the two-body excess entropy based on the pair correlation function accounts for about 90% of the configurational entropy, the local entropy of Zn atoms was calculated using the formula from Ref. [18] as follows:

$$S = -2\pi\rho k_{\mathrm{B}} \int_0^{r_m} [g(r) \ln g\,(r) - g(r) + 1] r^2 d\,r \quad (1)$$

$$g(r) = \frac{1}{4\pi r^2} \sum_j \frac{1}{\sqrt{2\pi\sigma^2}} e^{-\frac{(r-r_{ij})}{(2\sigma^2)}} \quad (2)$$

where ρ is the system density, k_B is the Boltzmann constant, r_m is the upper limit for integration, $g(r)$ is the radial function centered at the ith atom, r_{ij} is the distance between ith and jth atoms, and σ is the broadening parameter. Here, r_m was selected to be 5 Å, and σ to be 0.2. To increase the statistics of the analysis, the entropy calculations were based on the trajectory of equilibration.

Ring orientation index of the imidazole rings. The ring orientation index (ROI) of the imidazole rings was calculated based on the stereographic projection method [19], which enables the mapping of the three-dimensional vectors at the surface of a sphere onto a plane. Specifically, we used the following steps to calculate ROI as shown schematically shown in Fig. 4: (1) After identifying all the imidazole rings, the positions of the ring members were used to calculate the normal vector of each ring; (2) A unit sphere was constructed to include all the normal vectors; (3) For each projected plane (i.e., *yz*-, *xy*-, and *xz*-planes), the unit sphere was divided into two equal hemispheres, while all the normal vectors were transformed at the surface of one hemisphere since each normal had two opposite vectors; (4) Each normal vector was connected with the pole of the opposite hemisphere, and the intersection point at the projected plane was defined as the stereographic projection of the corresponding normal vector; and (5) The pole figure of each projected plane was obtained by calculating the local density of the points, while the distribution

histogram of each pole figure was calculated by binning the number of the points as a function of the central angle θ. Here, the bin size to calculate the histogram was selected as 2°. We note that the selection of bin size influences the value of ROI without changing the relative correlation (Supplementary Fig. S27). This analysis was also carried out on the trajectory of equilibration to increase statistics. The ROI value of each system was then finally calculated as,

$$\mathrm{ROI} = \frac{\int_0^{360} |h(x) - \langle h(x) \rangle| dx}{\int_0^{360} h(x) dx} \quad (3)$$

where $h(x)$ is the distribution histogram. The overall ROI value was obtained by averaging over the three projected planes. We note that the ROI is found not to be influenced by the selected system size as the pole figure is irrespective of the simulated system size (see Supplementary Fig. S17).

Supplementary Figures

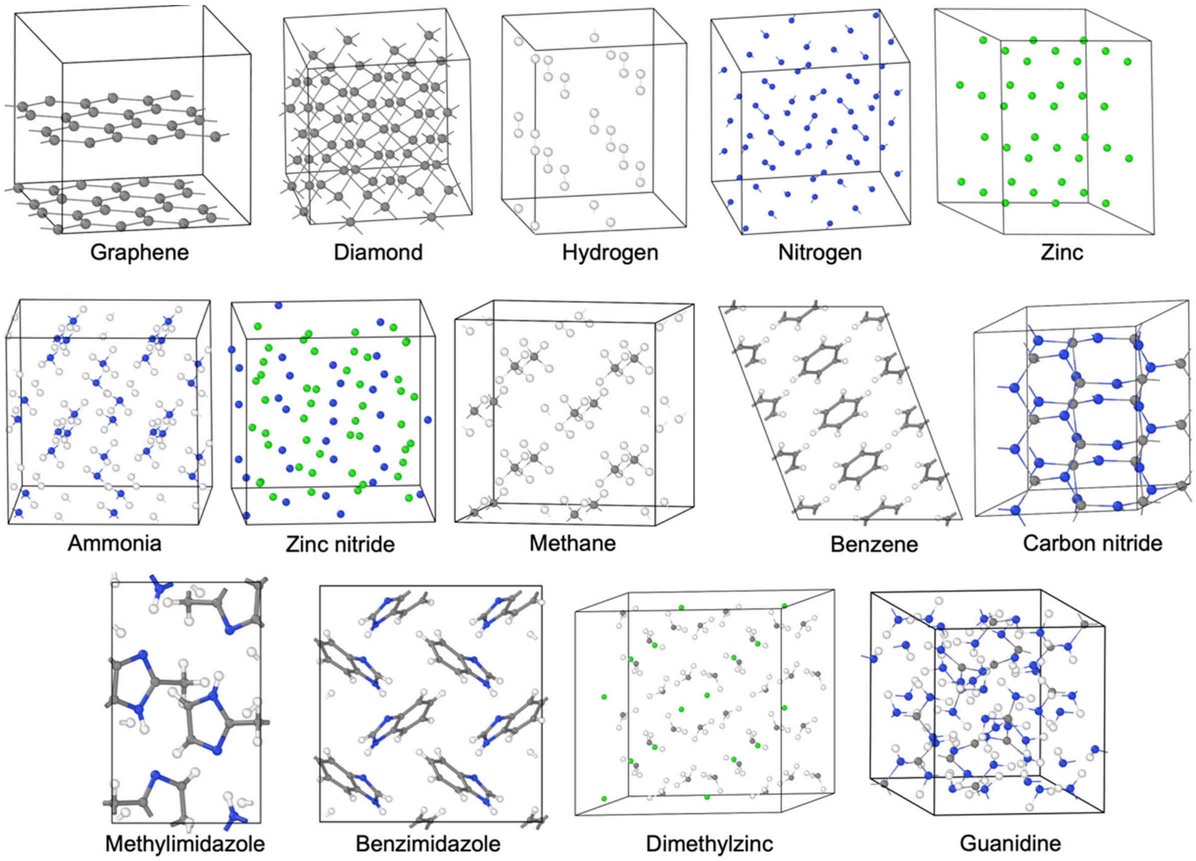

Figure S1. Selected configurations of atomic structures containing a subset of C, H, N, and Zn elements. Zn, C, H, and N are colored green, grey, white, and blue, respectively.

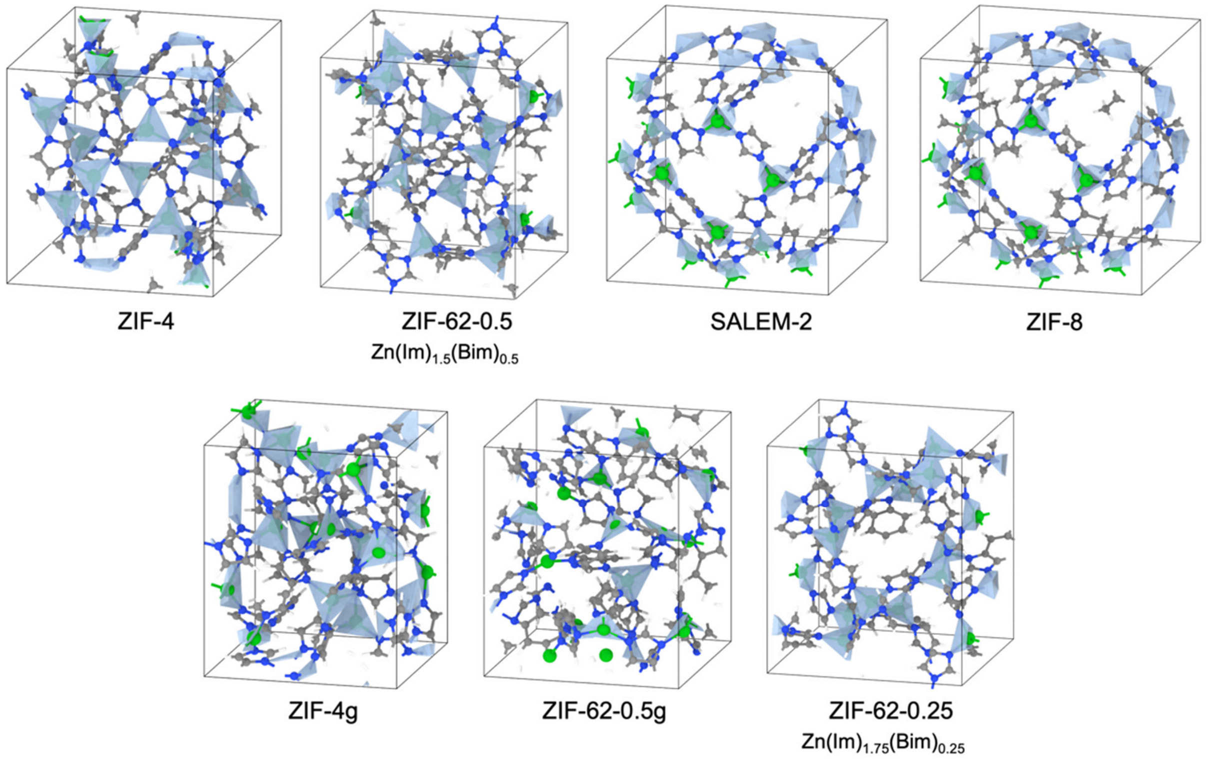

Figure S2. Selected configurations of atomic structures containing C, H, N, and Zn elements for the ab initio simulations. Zn, C, H, and N are colored green, grey, white, and blue, respectively. The four crystalline structures on the top are also used as the initial structures for the ab initio simulations after perturbation and active learning simulations using DPGEN.

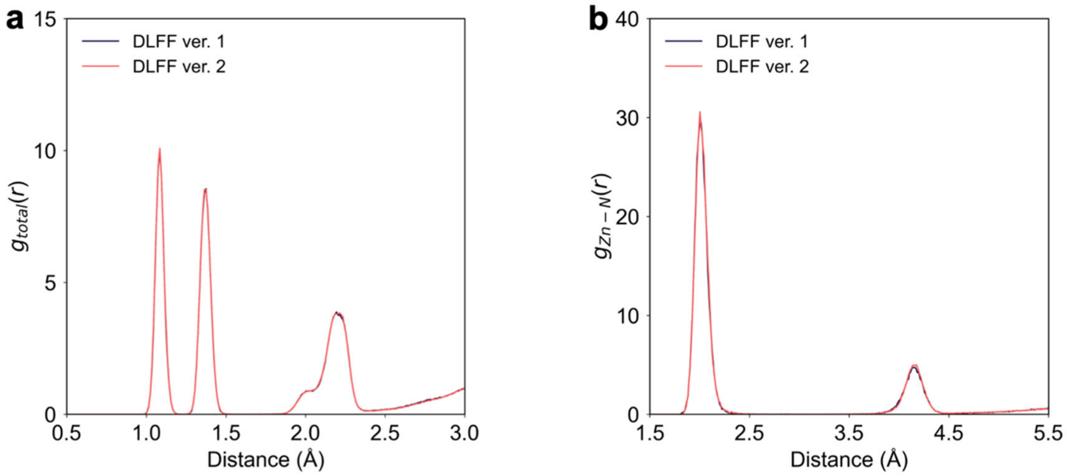

Figure S3. (a) Total and (b) Zn-N partial pair distribution functions of ZIF-4 glass from the melt quench simulation using the DLFF trained from different datasets. DLFF ver. 1 (the present datasets), and DLFF ver. 2 (extended datasets). Specifically, the extended datasets include the present datasets and additional datasets of the SALEM-2 crystal with methane molecules.

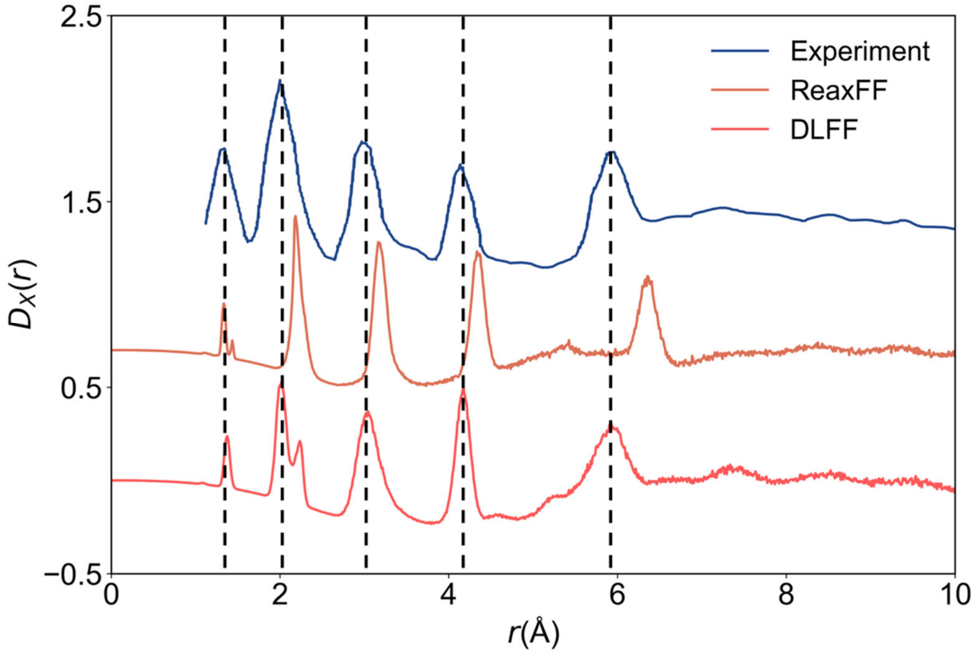

Figure S4. Comparison of experimental (X-ray) and MD-simulated differential correlation functions $D(r)$ of ZIF-4 glass using ReaxFF and DLFF. The experimental data is from Ref.[20].

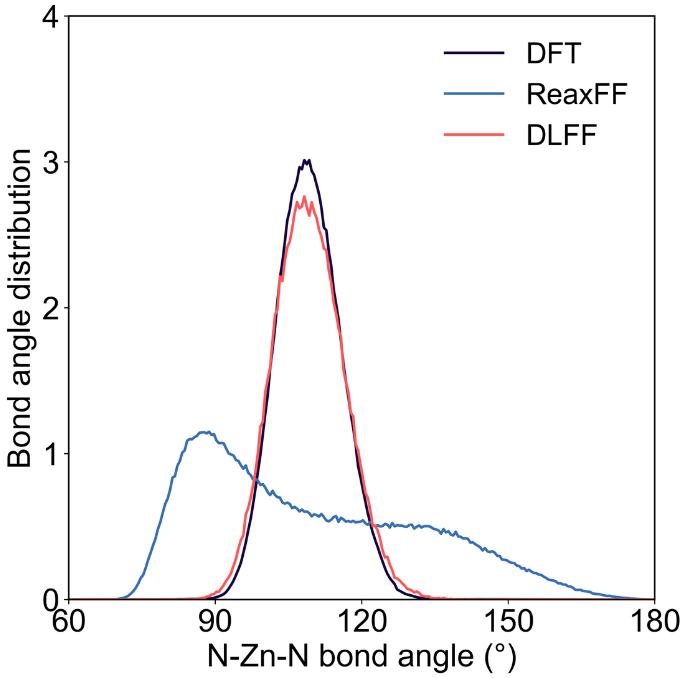

Figure S5. Distribution of N-Zn-N bond angle of ZIF-4 crystal simulated by density function theory (DFT), reactive force field (ReaxFF), and deep learning-based force field (DLFF).

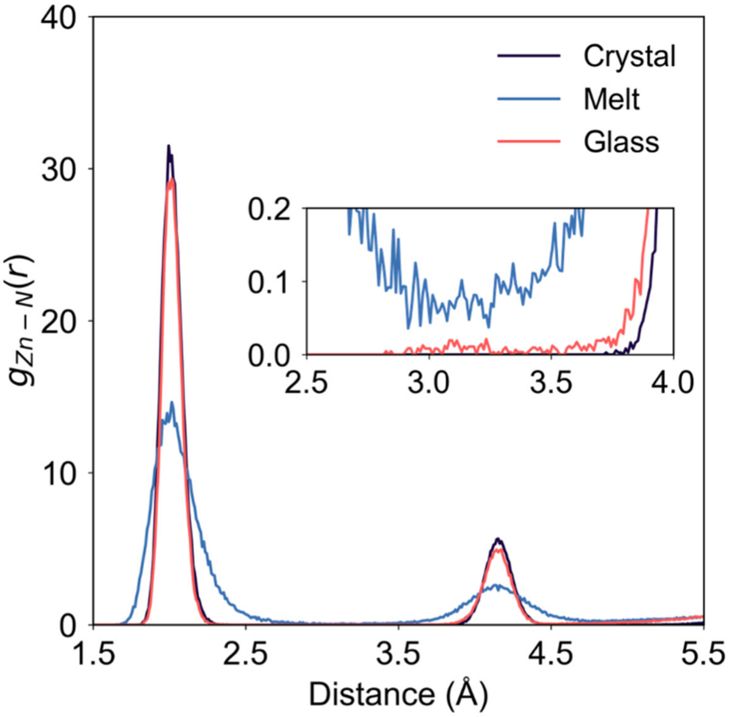

Figure S6. Zn-N partial pair distribution functions of ZIF-4 in crystalline, melt, and glassy states. Inset: Partial magnification to highlight the overlapped peaks in the molten state.

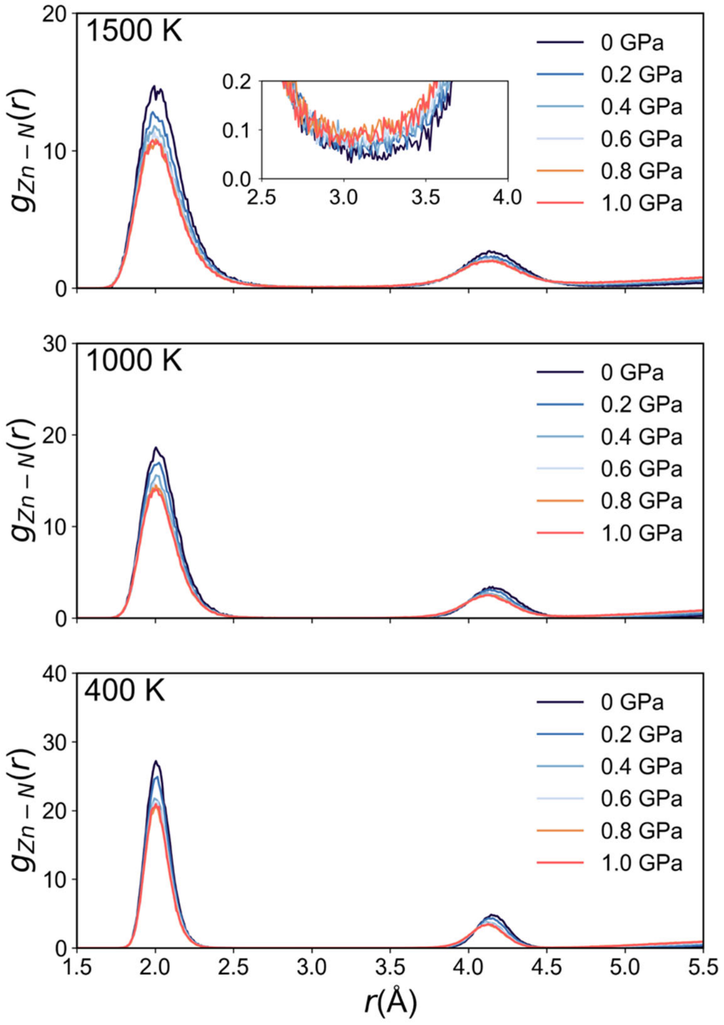

Figure S7. Zn-N partial pair distribution functions of ZIF-4 during the melting process of 400 K, 1000 K, and 1500 K at varying pressures (from 0 to 1.0 Gpa). Inset: Partial magnification to highlight the overlapped peaks.

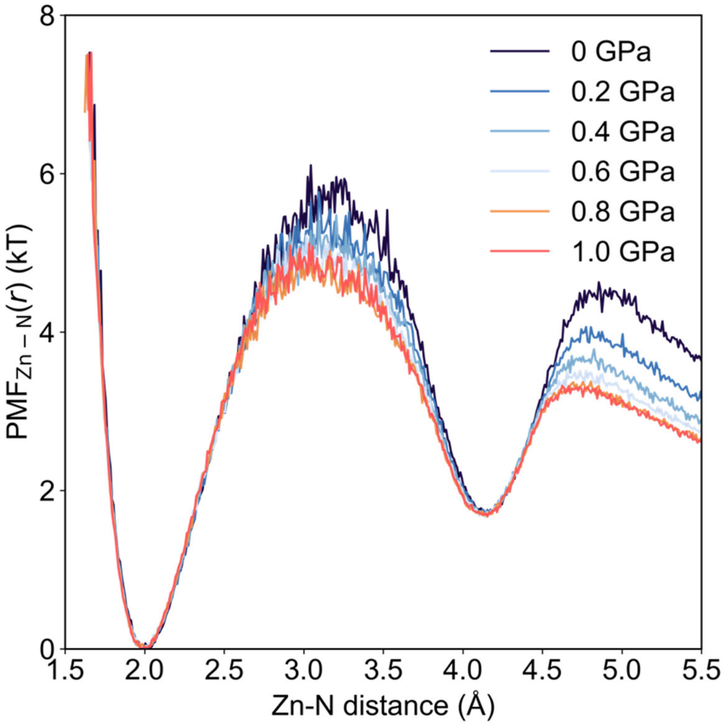

Figure S8. Potentials of mean force along the Zn−N coordinate for the structure at 1500 K and varying pressures in the unit of kT.

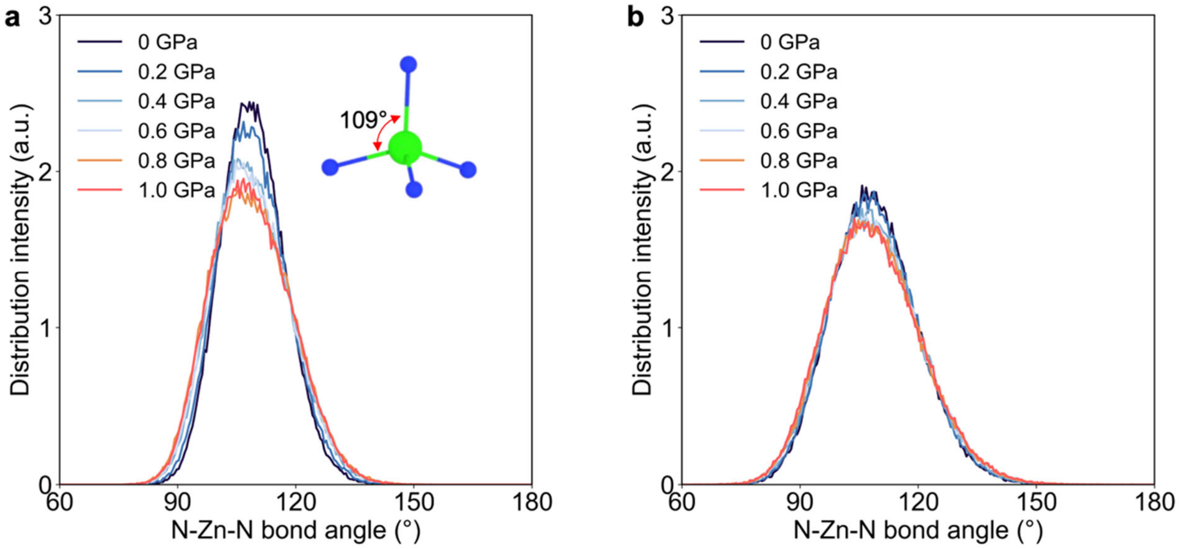

Figure S9. Distribution of N-Zn-N bond angle of (a) ZIF-4 at 400 K and (b) ZIF-4 at 1000 K under varying pressures.

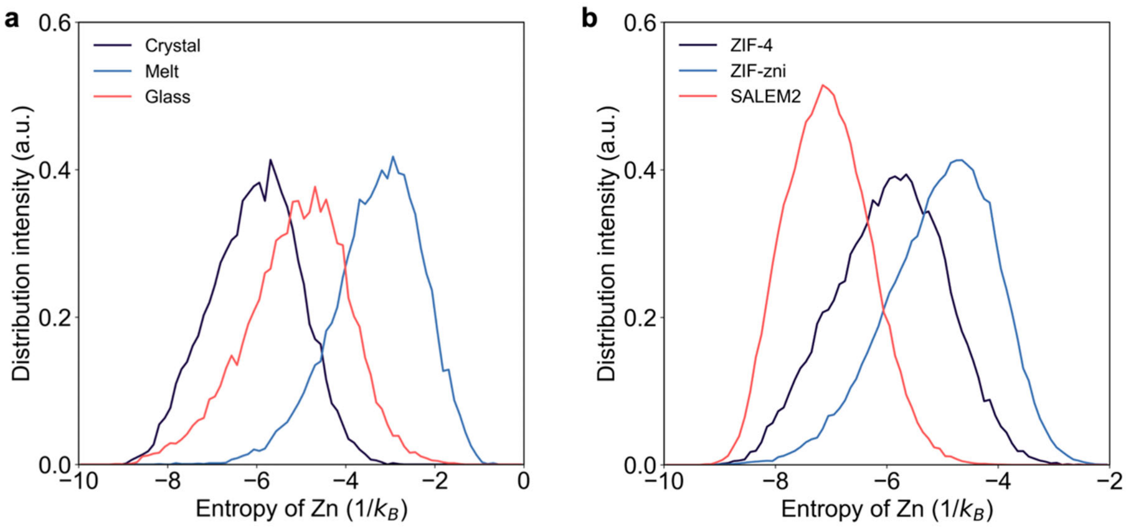

Figure S10. Distribution of entropy of Zn atoms in (a) ZIF-4 in crystalline, melt, and glassy states processed under zero pressure, and (b) different crystalline structures of $Zn(Im)_2$.

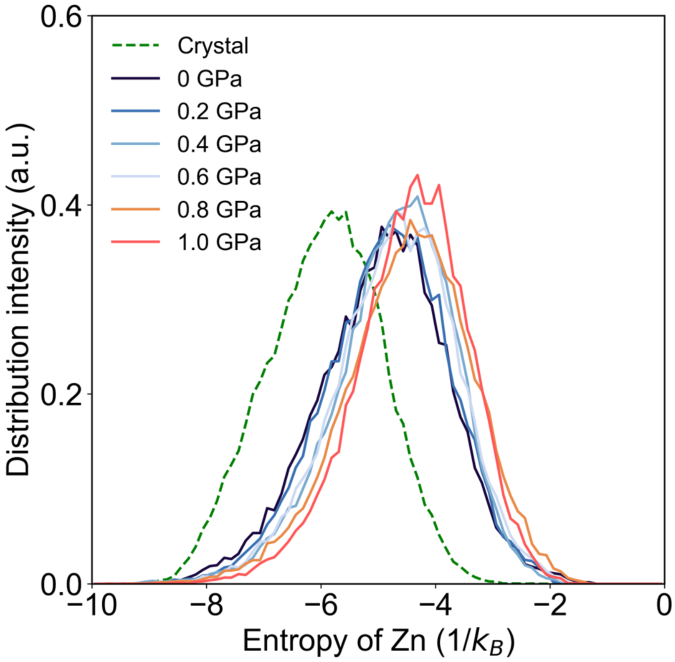

Figure S11. Distribution of entropy of Zn atoms in ZIF-4 glasses processed under varying pressures. For comparison, the entropy distribution of ZIF-4 crystal equilibrated at 300 K is also provided as the green dashed line.

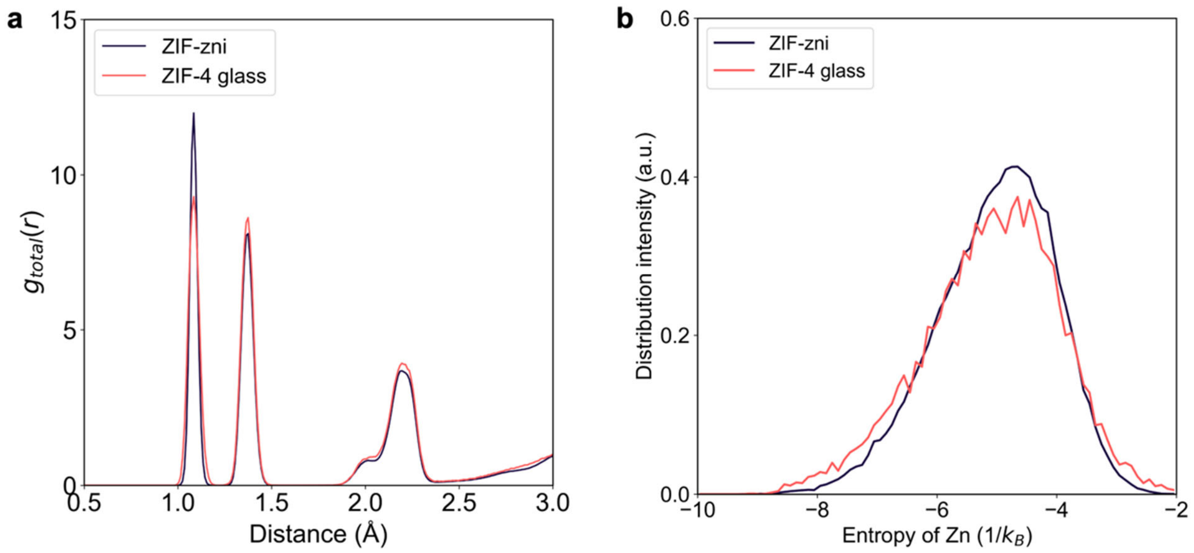

Figure S12. Comparison of structural properties of ZIF-zni crystal and ZIF-4 glass prepared under zero pressure. (a) Total pair distribution function. (b) Distribution of entropy of Zn atoms

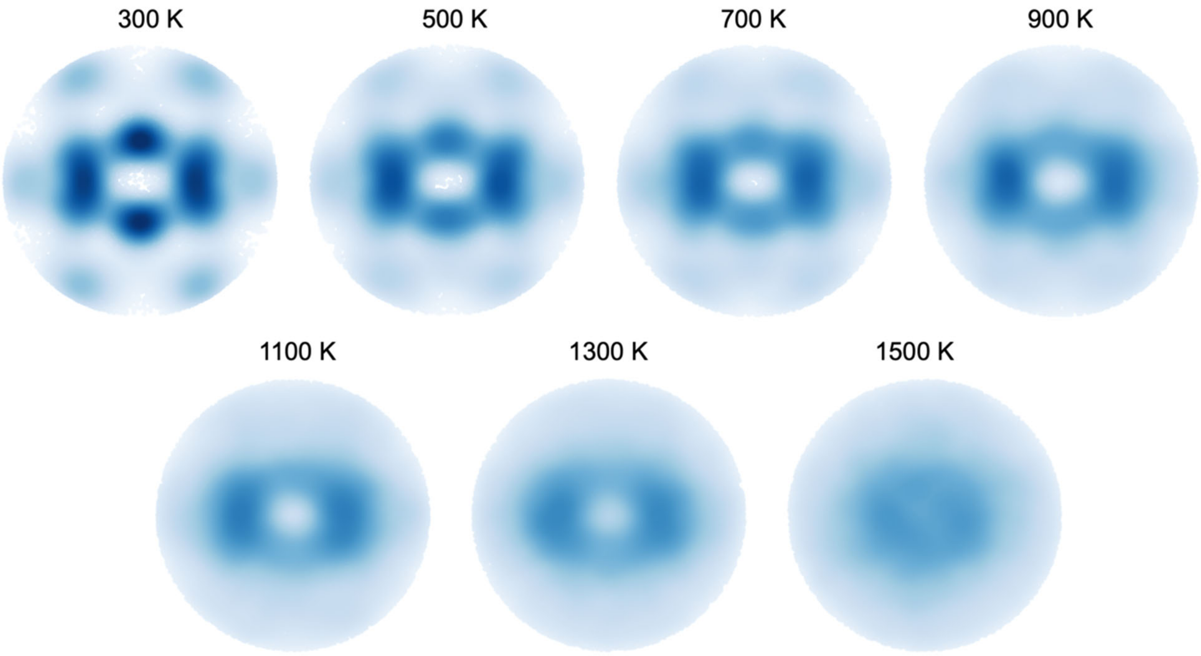

Figure S13. Pole figures of ring orientation projected on the *yz*-plane of ZIF-4 crystal melted at different temperatures under zero pressure.

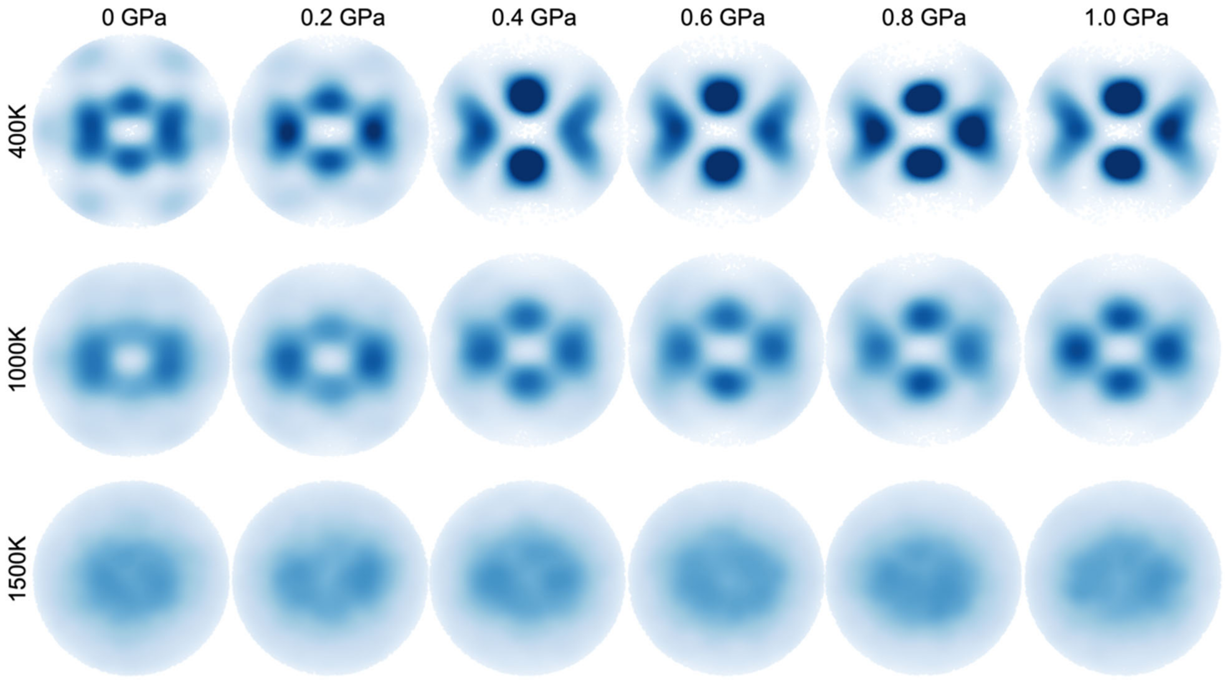

Figure S14. Pole figures of ring-normals for ZIF-4 at 400 K, 1000 K, and 1500 K under different pressures.

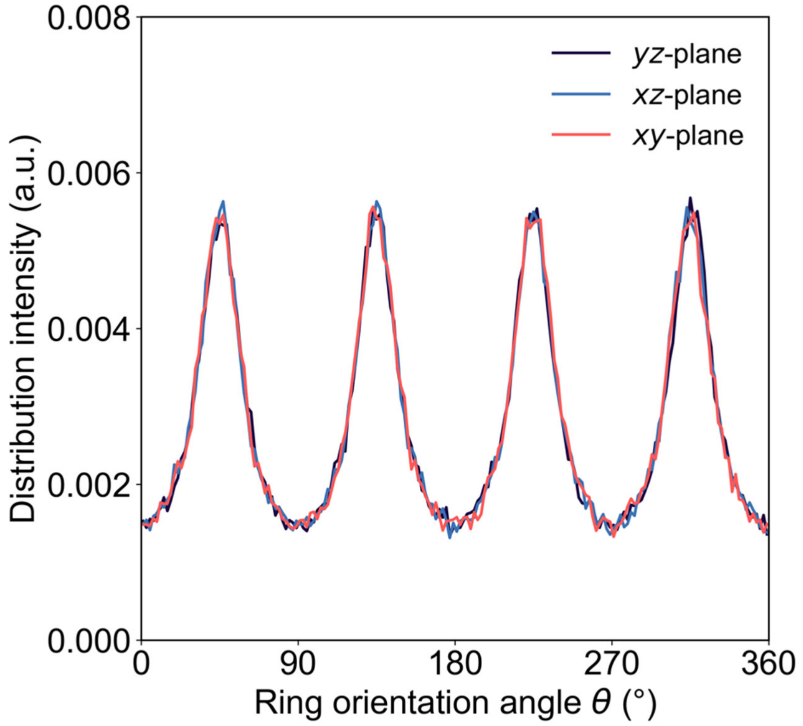

Figure S15. Orientation distribution histograms of imidazole ring in SALEM-2 crystal projected in all three directions.

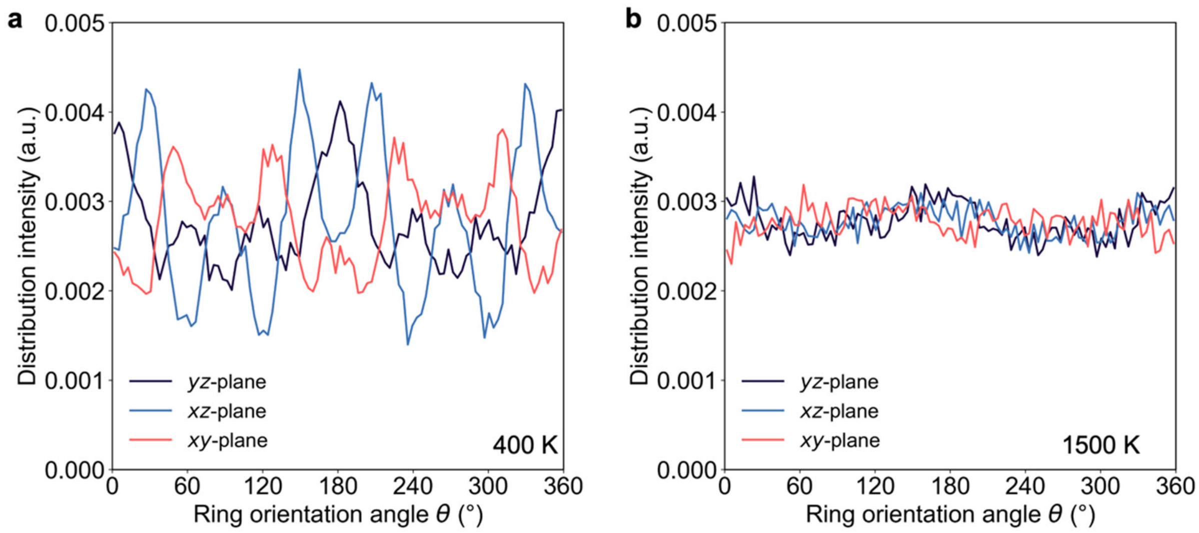

Figure S16. Orientation distribution histograms of imidazole ring in ZIF-4 at (b) 400 K and zero pressure and (c) 1500 K and zero pressure projected in all three directions.

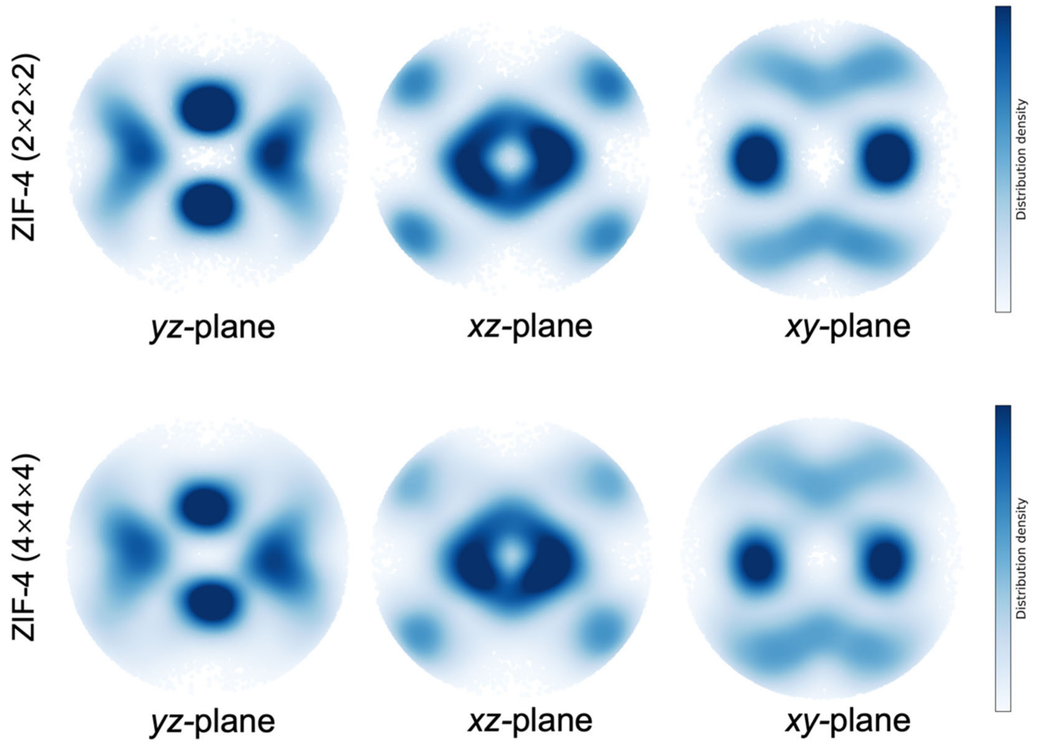

Figure S17. Pole figures of ring orientation of ZIF-4 crystal with different system sizes (2×2×2 vs. 4×4×4 supercells) melted at 400 K and 1 GPa.

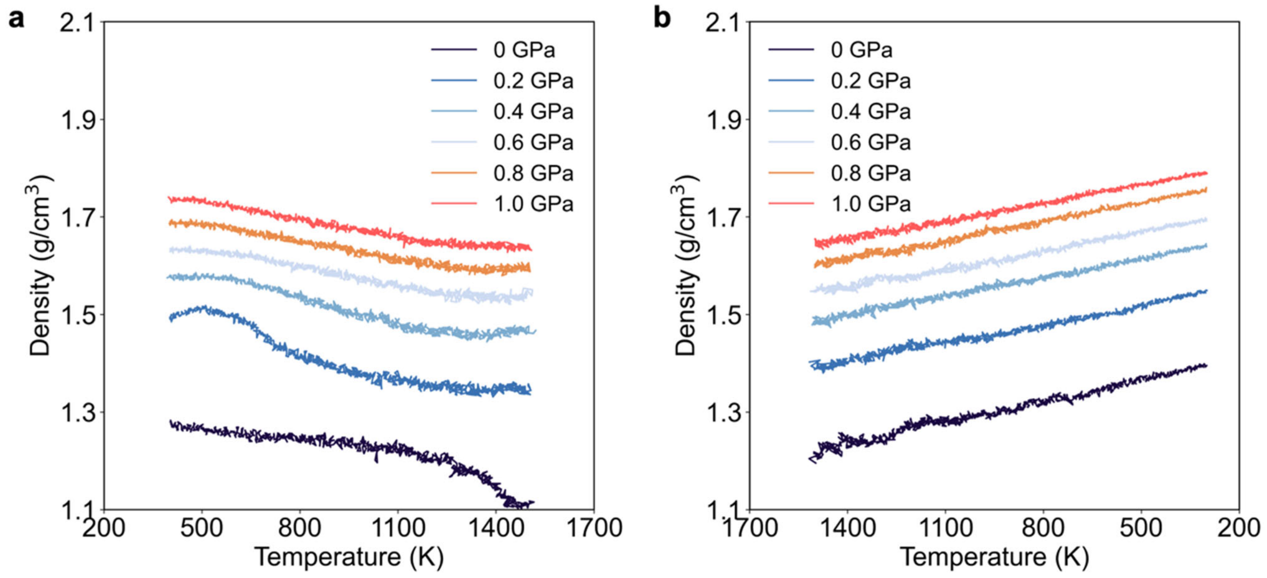

Figure S18. The density evolution as a function of temperature during (a) melting and (b) quenching process under varying pressures.

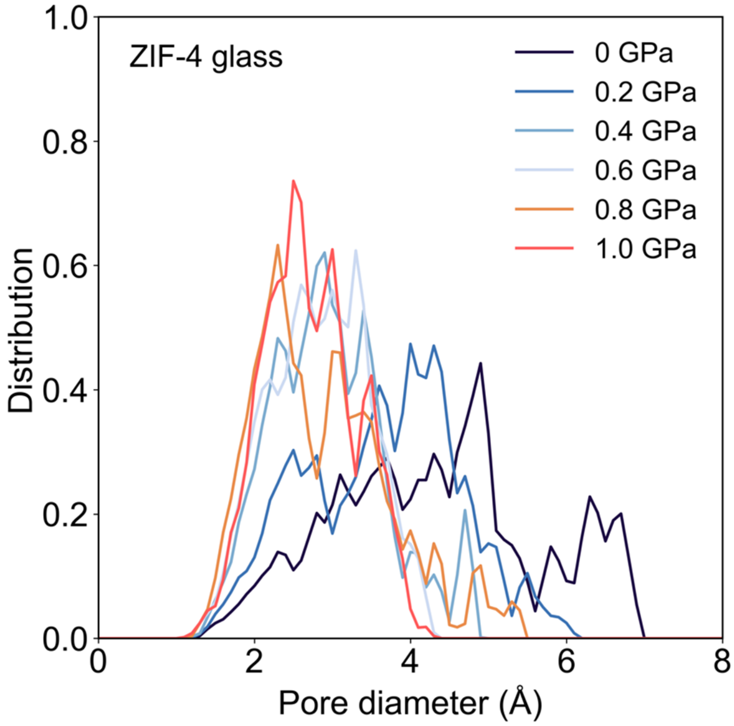

Figure S19. Pore size distributions of the ZIF-4 glasses prepared under varying pressures.

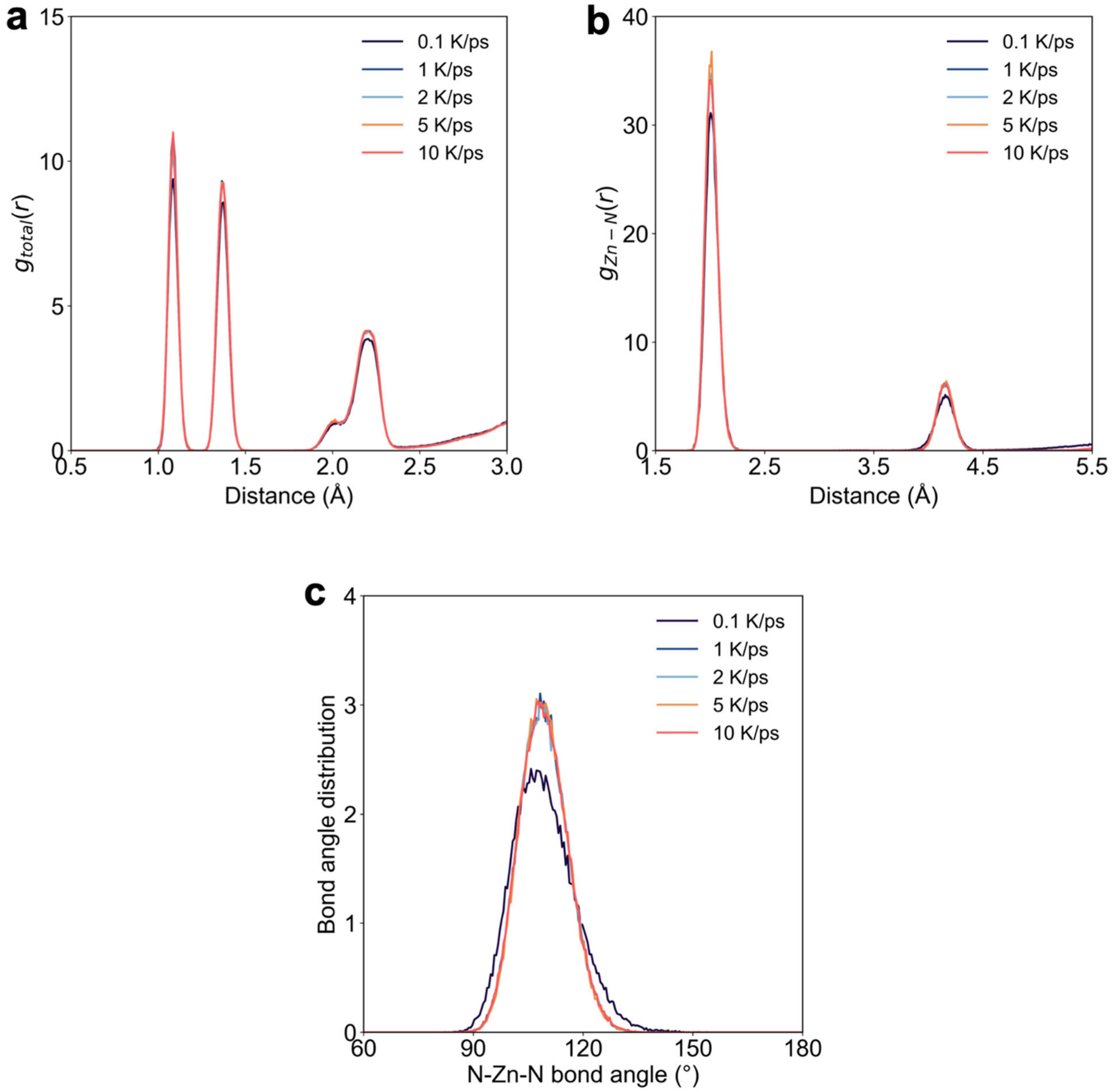

Figure S20. (a) Total and (b) Zn-N partial pair distribution functions, and (c) N-Zn-N bond angle distribution function of ZIF-4 glass quenched under different cooling rates.

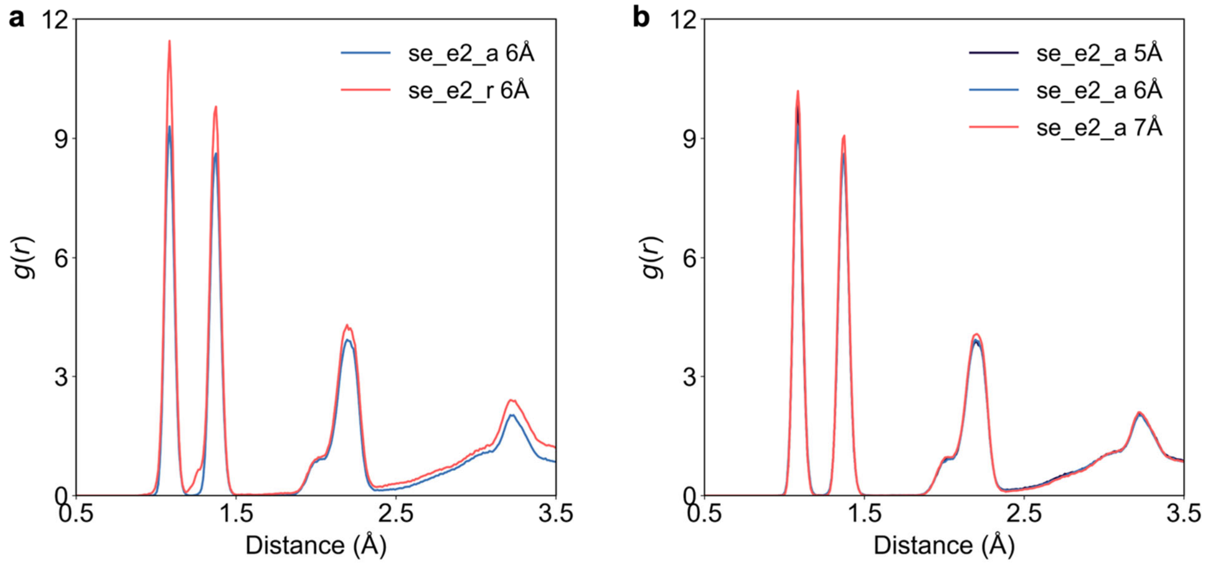

Figure S21. Pair distribution functions of the ZIF-4 glasses simulated using the DLFF with different models. (a) The "se_e2_a" and "se_e2_r" descriptors with a cutoff of 6 Å. (b) The "se_e2_a" descriptor with different cutoffs.

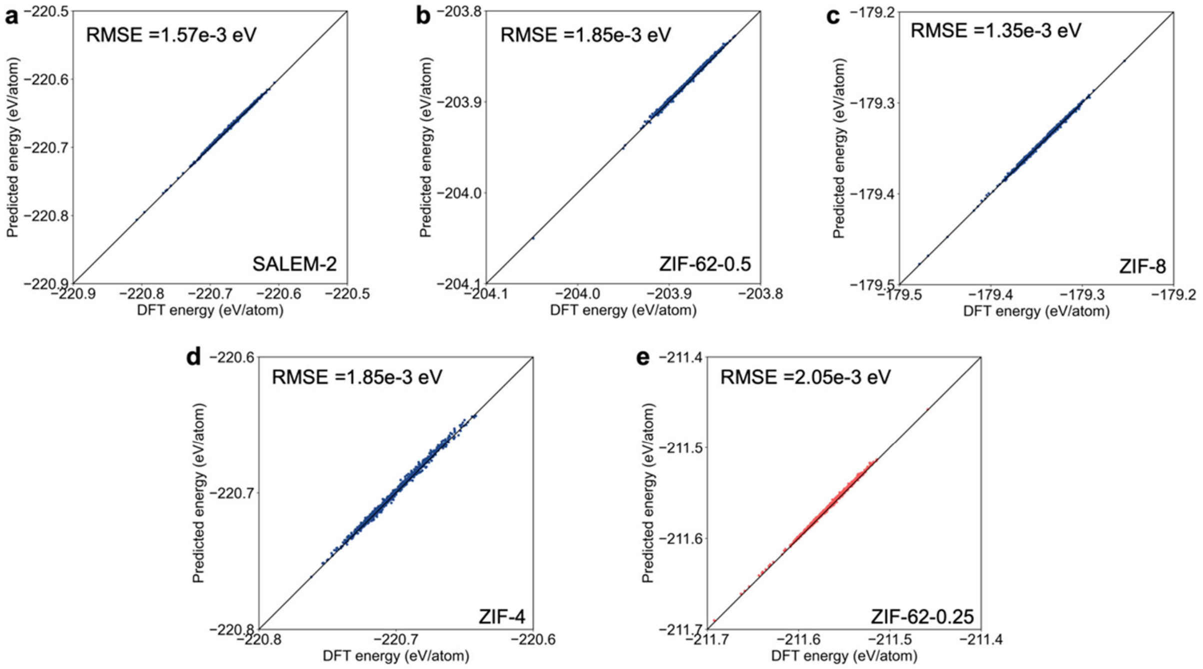

Figure S22. Comparison of the energy of different ZIF structures calculated by DFT and predicted by the present ML force field for both training sets of (a) SALEM-2, (b) ZIF-62-0.25, (c) ZIF-8, and (d) ZIF-4, and (e) test sets of ZIF-62-0.25.

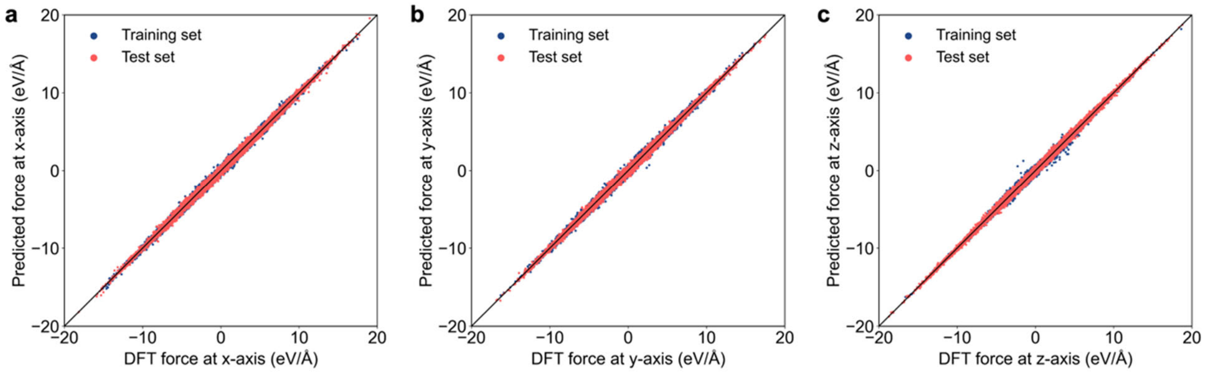

Figure S23. Comparison of the atomic force components of the unit cell of ZIF-62 calculated by DFT and predicted by the present ML force field for both training and test sets at (a) *x*-direction, (b) *y*-direction, and (c) *z*-direction.

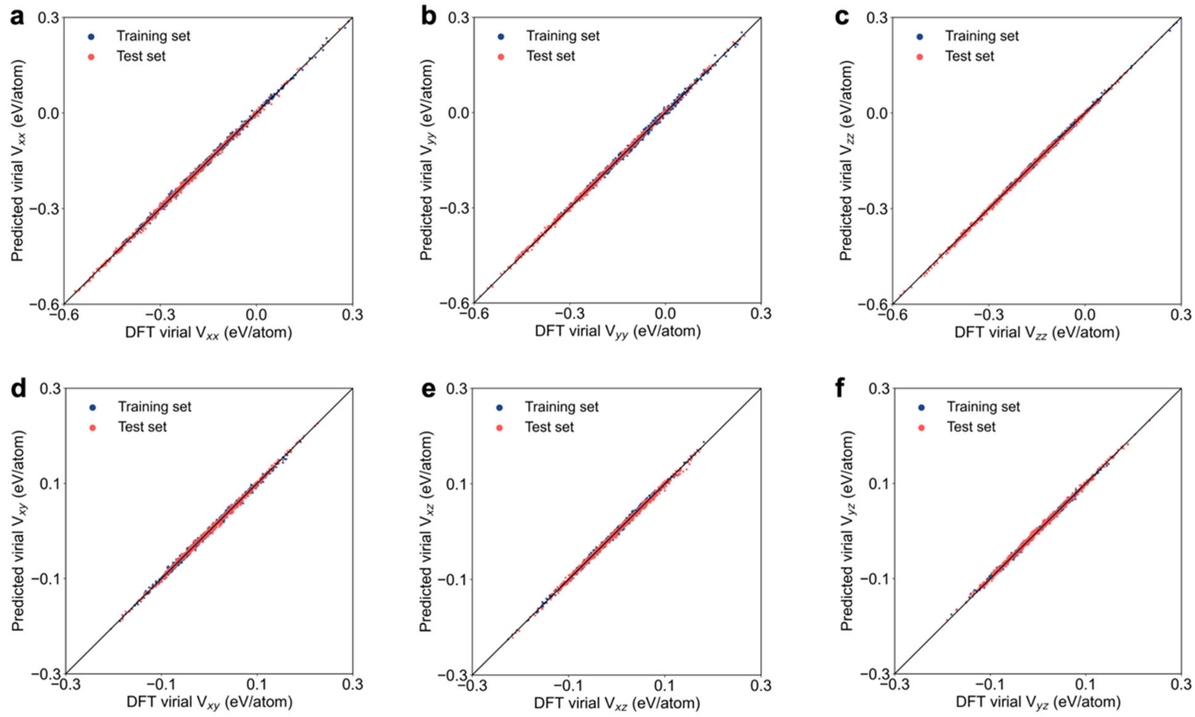

Figure S24. Comparison of the stress components of the unit cell of ZIF-62 calculated by DFT and predicted by the present ML force field for both training and test sets. (a) *xx*, (b) *yy*, (c) *zz*, (d) *x*€(e) *xz*, and (f) *yz* components.

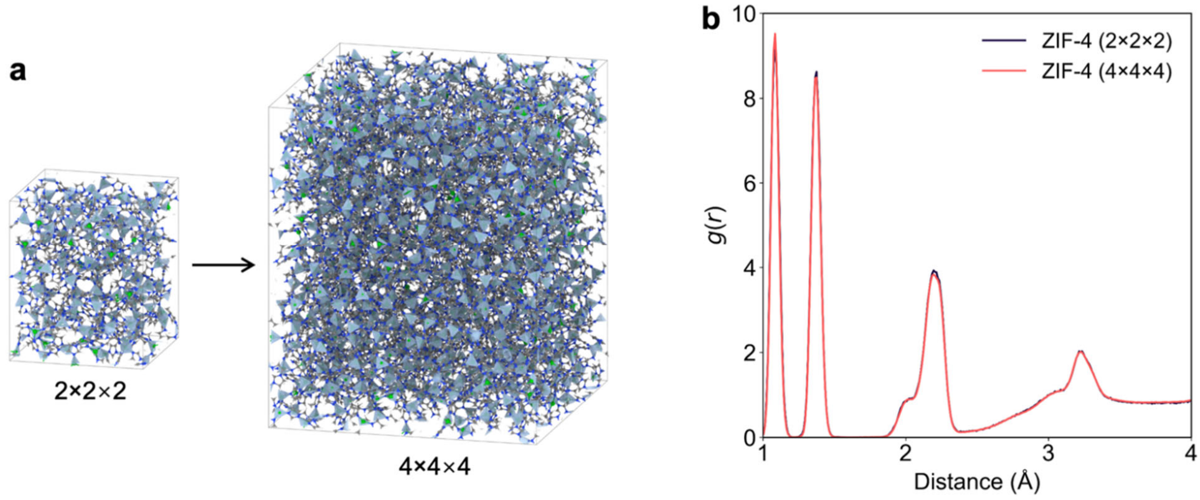

Figure S25. Simulated glass structure of ZIF-4 with different system sizes. (a) Atomic snapshots of ZIF-4 with system size of a 2×2×2 (2,176 atoms) and 4×4×4 (17,408 atoms) supercell. (b) Pair distribution function of ZIF-4 glass with different system sizes.

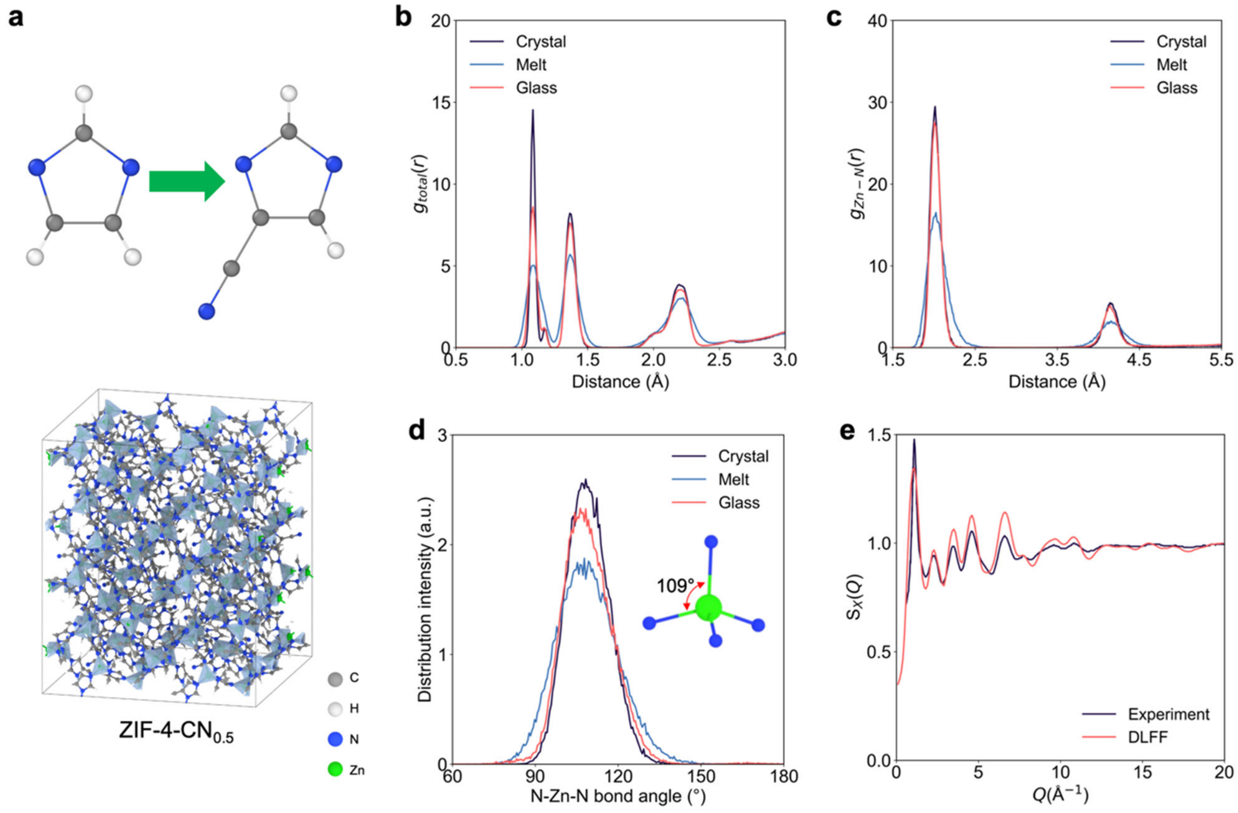

Figure S26. Application of the new DLFF to simulate the melt-quenching process of ZIF-4 with cyano-functionalized imidazolate linkers (CNim, 4-cynanoimidazolate). (a) Atomic snapshots of the cyano-functionalized imidazolate linker and ZIF-4-CN0.5 structure (x = 0.5 of composition $Zn(Im)_{2-x}(CNim)_x$. (b-d) Calculated (b) pair distribution functions, (c) Zn-N partial pair distribution function, (d) N-Zn-N bond angle distribution functions of the ZIF-4-CN0.5 crystal, melt (at 1300 K), and glass. (e) Simulated X-ray structure factor of ZIF-4-CN0.5 glass in comparison with experimental data. The atomic structure and experimental X-ray structure factor are adopted from Ref. [16].

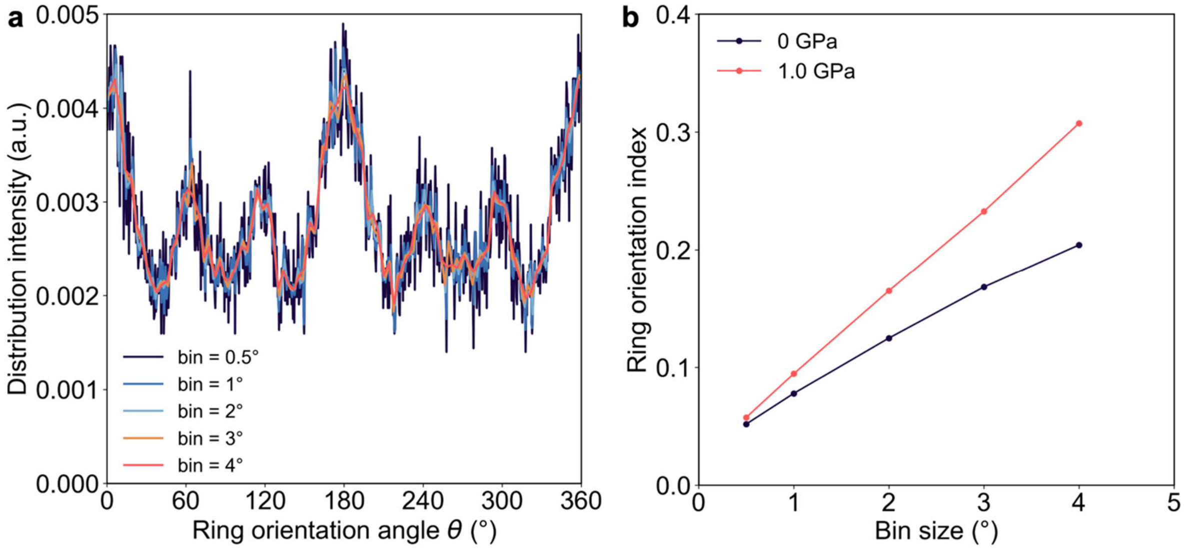

Figure S27. Calculated (a) ring orientation distribution histogram and (b) ring orientation index as a function of the bin size of the ring orientation distribution histogram.

Supplementary Tables

Table S1. Details of the initial data set obtained from the *ab initio* MD simulations.

System	Composition	Atom number	Number of configurations
Benzene	C6H6	192	1000
Benzimidazole	C7H6N2	240	1000
Methane	CH4	80	1000
Methylimidazole	C4H6N2	96	1000
Ammonia	NH3	128	1000
SALEM-2	C6N4H6Zn	204	1000
ZIF-4	C6N4H6Zn	272	1000
ZIF-4 glass	C6N4H6Zn	272	1000
ZIF-8	C8H10N4Zn	276	1000
ZIF-62	C8H7N4Zn	320	1000
ZIF-62 glass	C8H7N4Zn	320	1000
Zinc nitride	Zn3N2	80	1000
Dimethylzinc	C2H6Zn	144	1000
Diamond	C	64	1000
Graphene	C	48	1000
Hydrogen	H	32	1000
Nitrogen	N	64	1000
Zinc	Zn	36	1000

Table S2. Details of the explored data set obtained from the single energy calculation using CP2K.

Iteration	System	Composition	Atom number	Conditions	Number of configurations
1	ZIF-4	C6N4H6Zn	272	Temp(K): [500,1000,1500,2000] Press(bar): [0]	445
	ZIF-62	C8H7N4Zn	320		906
	ZIF-8	C8H10N4Zn	276		904
	SALEM-2	C6N4H6Zn	204		487
2	ZIF-4	ZnC6N4H6	272	Temp(K): [500,1000,1500,2000] Press(bar): [1]	1000
	ZIF-62	C8H7N4Zn	320		999
	ZIF-8	C8H10N4Zn	276		999
	SALEM-2	ZnC6N4H6	204		999
3	ZIF-4	ZnC6N4H6	272	Temp(K): [500,1000,1500,2000] Press(bar): [100]	1000
	ZIF-62	C8H7N4Zn	320		1000
	ZIF-8	C8H10N4Zn	276		998
	SALEM-2	ZnC6N4H6	204		997
4	ZIF-4	ZnC6N4H6	272	Temp(K): [500,1000,1500,2000] Press(bar): [500]	168
	ZIF-62	C8H7N4Zn	320		650
	ZIF-8	C8H10N4Zn	276		454
	SALEM-2	ZnC6N4H6	204		131
5	ZIF-4	ZnC6N4H6	272	Temp(K): [500,1000,1500,2000] Press(bar): [1000]	766
	ZIF-62	C8H7N4Zn	320		655
	ZIF-8	C8H10N4Zn	276		617
	SALEM-2	ZnC6N4H6	204		819
6	ZIF-4	ZnC6N4H6	272	Temp(K): [500,1000,1500,2000] Press(bar): [5000]	460
	ZIF-62	C8H7N4Zn	320		665
	ZIF-8	C8H10N4Zn	276		783
	SALEM-2	ZnC6N4H6	204		425
7	ZIF-4	ZnC6N4H6	272	Temp(K): [500,1000,1500,2000] Press(bar): [10000]	535
	ZIF-62	C8H7N4Zn	320		599
	ZIF-8	C8H10N4Zn	276		751
	SALEM-2	ZnC6N4H6	204		500
8	ZIF-4	ZnC6N4H6	204	Temp(K): [300,500,1000,1500] Press(bar): [0]	208
	ZIF-62	C8H7N4Zn	272		216
	ZIF-8	C8H10N4Zn	276		397
	SALEM-2	ZnC6N4H6	320		357

Supplementary References

1. Gaillac R, Pullumbi P, Bennett TD *et al.* Structure of Metal–Organic Framework Glasses by Ab Initio Molecular Dynamics. *Chem Mater* 2020;**32**:8004–11.
2. Kohn W, Sham LJ. Self-Consistent Equations Including Exchange and Correlation Effects. *Phys Rev* 1965;**140**:A1133–8.
3. Kühne TD, Iannuzzi M, Del Ben M *et al.* CP2K: An electronic structure and molecular dynamics software package - Quickstep: Efficient and accurate electronic structure calculations. *J Chem Phys* 2020;**152**:194103.
4. VandeVondele J, Krack M, Mohamed F *et al.* Quickstep: Fast and accurate density functional calculations using a mixed Gaussian and plane waves approach. *Computer Physics Communications* 2005;**167**:103–28.
5. Gaillac R, Pullumbi P, Coudert F-X. Melting of Zeolitic Imidazolate Frameworks with Different Topologies: Insight from First-Principles Molecular Dynamics. *J Phys Chem C* 2018;**122**:6730–6.
6. Nosé S. A molecular dynamics method for simulations in the canonical ensemble. *Molecular Physics* 1984;**52**:255–68.
7. Perdew JP, Burke K, Ernzerhof M. Generalized Gradient Approximation Made Simple. *Phys Rev Lett* 1996;**77**:3865–8.
8. Grimme S, Antony J, Ehrlich S *et al.* A consistent and accurate ab initio parametrization of density functional dispersion correction (DFT-D) for the 94 elements H-Pu. *J Chem Phys* 2010;**132**:154104.
9. Goedecker S, Teter M, Hutter J. Separable dual-space Gaussian pseudopotentials. *Phys Rev B* 1996;**54**:1703–10.
10. Zhang Y, Wang H, Chen W *et al.* DP-GEN: A concurrent learning platform for the generation of reliable deep learning based potential energy models. *Computer Physics Communications* 2020;**253**:107206.
11. Zeng J, Cao L, Xu M *et al.* Complex reaction processes in combustion unraveled by neural network-based molecular dynamics simulation. *Nat Commun* 2020;**11**:5713.
12. Zhou Y, Zheng H, Li W *et al.* A deep learning potential applied in tobermorite phases and extended to calcium silicate hydrates. *Cement and Concrete Research* 2022;**152**:106685.
13. Allen FH. The Cambridge Structural Database: a quarter of a million crystal structures and rising. *Acta Cryst B* 2002;**58**:380–8.
14. Qiao A, Sørensen SS, Stepniewska M *et al.* Hypersensitivity of the Glass Transition to Pressure History in a Metal–Organic Framework Glass. *Chem Mater* 2022;**34**:5030–8.
15. Yang Y, Shin YK, Li S *et al.* Enabling Computational Design of ZIFs Using ReaxFF. *J Phys Chem B* 2018;**122**:9616–24.
16. Song J, Frentzel-Beyme L, Pallach R *et al.* Modulating Liquid–Liquid Transitions and Glass Formation in Zeolitic Imidazolate Frameworks by Decoration with Electron-Withdrawing Cyano Groups. *J Am Chem Soc* 2023;**145**:9273–84.
17. Stukowski A. Visualization and analysis of atomistic simulation data with OVITO–the Open Visualization Tool. *Modelling Simul Mater Sci Eng* 2009;**18**:015012.
18. Piaggi PM, Parrinello M. Entropy based fingerprint for local crystalline order. *J Chem Phys* 2017;**147**:114112.
19. Ganisetti S, Atila A, Guénolé J *et al.* The origin of deformation induced topological anisotropy in silica glass. *Acta Materialia* 2023;**257**:119108.
20. Bennett TD, Yue Y, Li P *et al.* Melt-Quenched Glasses of Metal–Organic Frameworks. *J Am Chem Soc* 2016;**138**:3484–92.
